# Maackiain, a compound derived from *Sophora flavescens*, increases IL‐1β production by amplifying nigericin‐mediated inflammasome activation

**DOI:** 10.1002/2211-5463.12899

**Published:** 2020-06-26

**Authors:** Jin‐Won Huh, Jung‐Hoon Lee, Eekhyoung Jeon, Hyung Won Ryu, Sei‐Ryang Oh, Kyung‐Seop Ahn, Hyun Sik Jun, Un‐Hwan Ha

**Affiliations:** ^1^ Department of Biotechnology and Bioinformatics Korea University Sejong Korea; ^2^ Natural Medicine Research Center Korea Research Institute of Bioscience and Biotechnology Chungbuk Korea

**Keywords:** (‐)‐maackiain, inflammasome, monophosphoryl lipid A, nigericin, *Sophora flavescens*

## Abstract

*Sophora flavescens* is used as a traditional herbal medicine to modulate inflammatory responses. However, little is known about the impact of (‐)‐maackiain, a compound derived from *S. flavescens*, on the activation of inflammasome/caspase‐1, a key factor in interleukin‐1β (IL‐1β) processing. Here, we report that (‐)‐maackiain potently amplified caspase‐1 cleavage in macrophages in response to nigericin (Nig). In macrophages primed with either lipopolysaccharide or monophosphoryl lipid A, Nig‐mediated caspase‐1 cleavage was also markedly promoted by (‐)‐maackiain. Notably, (‐)‐maackiain induced the production of vimentin, an essential mediator for the activation of the NOD‐, LRR‐, and pyrin domain‐containing protein 3 inflammasome, thereby contributing to promotion of the formation of the inflammasome complex to activate caspase‐1. Taken together, our data suggest that (‐)‐maackiain exerts an immunostimulatory effect by promoting IL‐1β production via activation of the inflammasome/caspase‐1 pathway. Thus, the potent inflammasome‐activating effect of (‐)‐maackiain may be clinically useful as an acute immune‐stimulating agent.

## Graphical abstract

Proinflammatory cytokine interleukin‐1β (IL‐1β) plays critical roles in defending against infections. *Sophora flavescens*‐derived (‐)‐maackiain potently amplifies caspase‐1 cleavage in response to nigericin. Notably, (‐)‐maackiain promotes caspase‐1 cleavage and IL‐1β production even in monophosphoryl lipid A‐primed macrophages. The potent inflammasome‐activating effect of (‐)‐maackiain may be clinically useful as an acute immune‐stimulating agent.

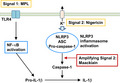

**Abbreviations**
alum: Aluminum salts
GSDMD: gasdermin D
IL‐1β: interleukin‐1β
LPS: lipopolysaccharide
MPL: monophosphoryl lipid A
NF‐κB: nuclear factor kappa B
NLRP3: NOD‐, LRR‐, and pyrin domain‐containing protein 3
TLR: Toll‐like receptor

In diverse cell types, including monocytes and macrophages, the key proinflammatory cytokine interleukin‐1β (IL‐1β) is produced by a two‐step process: an initial priming signal and a second activating signal [1]. Production of the pro‐form of IL‐1β is driven by nuclear factor kappa B (NF‐κB), which is activated by Toll‐like receptor (TLR) signaling in response to a priming signal, for example, pathogen‐associated molecular patterns including lipopolysaccharide (LPS). In response to the activating signal [e.g., nigericin (Nig), a toxin derived from *Streptomyces hygroscopicus* [2]], pro‐IL‐1β is cleaved to its active form by the inflammasome, a multiprotein complex comprising apoptosis‐associated speck‐like protein containing a CARD (ASC), NOD‐like receptor, and pro‐caspase‐1. Once the inflammasome is activated, pro‐caspase‐1 is cleaved to yield its enzymatically active heterodimer, consisting of subunits p10 and p20. The active caspase cleaves not only pro‐IL‐1β, but also gasdermin D (GSDMD), a caspase‐1 substrate that forms membrane pores [2, 3]. The cleaved form of GSDMD then facilitates the release of biologically active IL‐1β.

Released IL‐1β stimulates acute inflammatory responses involved in defense against microbial infections, including influenza [4]. Consistent with this, intranasal delivery of IL‐1β or mucosal delivery of *IL‐1β*‐encoding adenoviral vectors along with influenza vaccine boosts immune responses against influenza infections, demonstrating that its immune‐stimulatory effect is physiologically important [5, 6]. Elderly mice infected with influenza produce less amounts of active IL‐1β due to reductions in the levels of inflammasome‐related proteins, although they produce normal levels of pro‐IL‐1β [7]. This suggests that these animals have normal NF‐κB activation, but are deficient in stimulation of the inflammasome. Notably in this regard, the impairment in inflammasome activity is rescued to some extent by Nig treatment, which increases caspase‐1‐augmented IL‐1β production during influenza infection [7]. This observation implies that it could be clinically useful to amplify the activation of inflammasome/caspase‐1.

In East Asian countries, the root of *Sophora flavescens* is used as a traditional herbal medicine for the treatment of infectious diseases [8], cancer [9], and inflammatory disorders [10]. Typically, it is well known as a traditional antipyretic medicine by reducing inflammatory responses, and the anti‐inflammatory activity was proven by inhibiting the release of proinflammatory cytokines, including TNF‐α, IL‐6, and MCP‐1, on LPS‐induced RAW264.7 cells [11]. Given that *S. flavescens* and its preparations, such as Fufang Kushen Lotion and Fufang Kushen Injection, have been clinically used to treat the diseases [12, 13], phytochemical studies have shown that it contains obvious flavonoids with pleiotropic activities including anti‐inflammatory [14, 15]. In addition, (‐)‐maackiain, which was originally isolated from *Maackia amurensis var* [16], is widely distributed as a flavonoid analog in different plant genus including *Sophora* [17, 18], and its antimicrobial effects have been reported [17].

Previously, we reported that treatment with total *S. flavescens* extracts decreases *Pseudomonas aeruginosa*‐mediated expression of IL‐1β [19]. Unlike the anti‐inflammatory effects shown by the extract, we found the stimulating effect of (‐)‐maackiain, one of the compounds derived from *S. flavescens*, on the activation of inflammasome in macrophages following treatment with Nig, a microbial toxin that activates the NOD‐, LRR‐, and pyrin domain‐containing protein 3 (NLRP3) inflammasome. In these experiments, macrophages were first primed with either LPS or monophosphoryl lipid A (MPL), both of which activate key innate immune pathways through TLR4. The activating effect of (‐)‐maackiain was proven by the increase in caspase‐1 cleavage once treated with Nig, and this can lead to enhanced production of IL‐1β in either LPS‐ or MPL‐primed cells. In light of the physiological importance of inflammasome/caspase‐1 activation, our results highlight the potential use of (‐)‐maackiain to boost acute immune‐stimulatory responses.

## Materials and methods

### Reagents

Dried extract of *S. flavescens* and its derivative compounds were purchased from KPEB (Korea Plant Extract Bank, Cheongju, Republic of Korea; http://extract.kribb.re.kr). *P. aeruginosa*‐derived LPS, Nig, and acetyl‐tyrosyl‐valyl‐alanyl‐aspartyl‐chloromethylketone (ac‐YVAD‐cmk) were purchased from Sigma‐Aldrich (St. Louis, MO, USA). MPL and alum were purchased from InvivoGen (San Diego, CA, USA).

### Instruments

NMR spectra were recorded on a Bruker AM500 instrument (^1^H NMR at 500 MHz, ^13^C NMR at 125 MHz; Bruker, Karlsruhe, Germany), and tetramethylsilane was used as the internal standard. Chemical shifts are reported in p.p.m. (parts per million) and coupling constants (*J*) in Hz. Ultra‐performance liquid chromatography (UPLC) was performed using an ACQUITY UPLC™ system (Waters Corporation, Milford, MA, USA) equipped with a binary solvent delivery manager, a photodiode array (PDA). HRESIMS was performed on a Waters Q‐Tof Premier™ Mass Spectrometer equipped with an electrospray interface (Waters Corporation). Separations were conducted on an Armen Spot Prep II 250 medium‐pressure liquid chromatograph (MPLC) and on a PLC 2020 prep/high‐performance liquid chromatograph (prep‐HPLC; Gilson, Inc., Middleton, WI, USA) using an appropriately sized reversed‐phase silica gel column purchased from Waters. All solvents used for column chromatography were of analytical grade (SK Chemicals Co. Ltd., Seongnam, Korea), and all solvents used for HPLC were of HPLC grade (SK Chemicals Co., Ltd.). NMR solvents were purchased from Cambridge Isotope Laboratories Inc. (Andover, MA, USA).

### Extraction and isolation

*Sophora flavescens* was collected from Yeongwol‐gun, Gangwon‐do, Korea, in 2016. The plant (50 g) dried in the shade and powdered was added to 1 L of methanol (MeOH; HPLC Grade) with an ultrasonicator (SDN‐900H; SD Ultrasonic Cleaner, Seoul, Korea) at room temperature for 3 days (15‐min ultrasonication followed by 120‐min standing per cycle; repeated 30 cycles). After filtration and drying under reduced pressure, *S. flavescens* extract (5.63 g, 11.2%) was obtained. Crude extract (about 950 mg) was submitted to MPLC (Armen Spot Prep II 250; Gilson, Inc) reversed‐phase silica gel (YMC ODS‐AQ, 10 µm, 220 g) using a stepwise MeOH‐H_2_O gradient (35–100% MeOH, 20 mL·min^−1^, 90 min) to give nine fractions. Each of SF Fr.4 (117.6 mg), SF Fr.7 (99.6 mg), and SF Fr.8 (156.0 mg) was subjected to preparative chromatography using PLC2020 prep‐HPLC eluted with H_2_O‐MeOH gradient (30–60% MeOH) to yield 4‐hydroxy‐3‐methoxy‐8,9‐methylenedioxy pterocarpan (**7**; 8.8 mg), formononetin (**1**; 11.3 mg), (‐)‐maackiain (**5**; 8.5 mg), and sophoraflavanone B (**8**; 15.7 mg) from SF Fr.4; noranhydroicaritin (**6**; 10.4 mg), kushenol F (**4**; 37.5 mg), and (2S)‐2'‐methoxykurarinone (**2**; 80.5 mg) from SF Fr.7; and kushenol E (**3**; 13.2 mg) from SF Fr.8, respectively. Purities (> 98%) were confirmed by UPLC‐PDA‐Q/TOF‐M. Purified compounds were identified by comparing ^1^H NMR, ^13^C NMR, MS, MS/MS, HRESIMS, and optical rotation data with literature values (Supporting information).

### Bacterial culture and infection condition

*Pseudomonas aeruginosa* PAO1 wild‐type strain [20] was cultured in Luria (L) broth or on L agar plates at 37 °C. The cultured bacterial cells were harvested by centrifugation at 10 000 ***g*** for 20 min at 4 °C after overnight broth culture. The bacterial pellet was suspended in PBS for the preparation of live bacteria. Bacterial infection was performed according to the conditions previously described [19]. Briefly, cells were infected with *P. aeruginosa* strain PAO1 at a multiplicity of infection of 10 for 4 h.

### Cell culture and treatment condition

RAW‐Blue cells (mouse macrophage reporter cells; InvivoGen) were maintained in Dulbecco’s modified Eagle’s medium (containing high glucose, l‐glutamine, and sodium pyruvate; HyClone, Logan, UT, USA). A549 (human alveolar epithelial) and THP‐1 (human monocyte) cells were cultured in RPMI‐1640 (HyClone). Media supplemented with 10% heat‐inactivated FBS (Access, Vista, CA, USA), penicillin (100 units per mL), and streptomycin (0.1 mg·mL^−1^) were used to cultivate cells. Cells were maintained at 37 °C in a humidified 5% CO_2_ air‐jacketed incubator. Differentiation of THP‐1 cells (dTHP‐1) was achieved by the treatment with PMA (phorbol‐12‐myristate‐13‐acetate; 50 ng·mL^−1^) for 48 h followed by resting for 24 h in the presence of FBS. Unless otherwise indicated, cells were exposed to the following condition as described. For the assay of alkaline phosphatase activity, cells were pretreated with either *S. flavescens* extract (Ext; 50 ng·mL^−1^) or the derivative compounds (**1**‐**8**; 50 ng·mL^−1^) for the indicated time. For the stimulation of inflammasome activity, cells were primed by treatment with 100 ng·mL^−1^ of either LPS or MPL for 3 h. Then, cells were sequentially treated with Nig (5 µm) for 1 h and either (‐)‐maackiain (MK; 100 ng·mL^−1^) or alum (300 ng·mL^−1^) for 1 h.

### Alkaline phosphatase assay

RAW‐Blue cells are derived from RAW 264.7 macrophages with chromosomal integration of a secreted embryonic alkaline phosphatase (SEAP) reporter gene inducible by NF‐κB. Measurement of SEAP was performed according to the method previously described [19].

### Determination of cell viability

The cell culture media from component‐treated cells were analyzed for lactate dehydrogenase (LDH) release using a commercially available kit (CytoTox 96 Non‐Radioactive Cytotoxicity Assay; Promega, Madison, WI, USA) following the manufacturer’s instructions.

### Immunoblot analysis

Antibodies were used to analyze total cell lysates as per the manufacturers’ instructions. Antibodies of pro‐caspase‐1 (D7F10), caspase‐1 p20 (D57A2), pro‐IL‐1β (D3U3E), mature IL‐1β (D3U3E), cleaved GSDMD (L60), NLRP3 (D2P5E), NEK7 (C34C3), vimentin (D21H3), and β‐actin (D6A8) were purchased from Cell Signaling (Danvers, MA, USA). GSDMD (PA5‐30823) antibody was purchased from Thermo Scientific (Rockford, IL, USA). Immunoblot analysis was performed according to the method previously described [19].

### Cell ELISA

The level of IL‐1β in culture supernatants was determined by microplate sandwich ELISA using human IL‐1β ELISA Kits (Thermo, Rockford, IL, USA) following the manufacturer's instructions.

### Statistics

All experiments were carried out in triplicate. Results were expressed as mean ± SD. The Student *t*‐test was used to perform statistical analysis, with significance considered to be *P* < 0.05.

## Results

### Screening of *S. flavescens*‐derived compounds

**Fig. 1:**
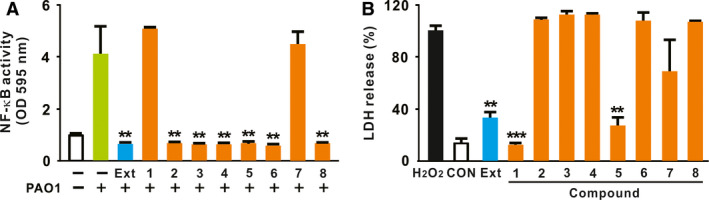
Screening of *S. flavescens*‐derived compounds. (A) RAW‐Blue cells were seeded in 12‐well plates at a concentration of 1 × 10^6^ cell per mL, and the cells were pretreated with either *S. flavescens* total Ext or the derivative compounds (**1**‐**8**) for 1 h. Cells were then infected with *P. aeruginosa* strain PAO1. After treatment, bacteria‐induced NF‐κB activation was measured by alkaline phosphatase assay. (B) A549 cells were seeded in 12‐well plates at a concentration of 2 × 10^5^ cell per mL, and the cells were treated with either total Ext or the derivative compounds (**1**‐**8**) for 24 h. After treatment, cytotoxicity was assessed by LDH release assay. Data are expressed as means ± SD (*n* = 3). Student’s *t*‐test: **P < 0.01, ***P < 0.001 *vs*. treatment with PAO1 alone (A) and H_2_O_2_ treatment (B). PBS was used as a control (CON). Compounds: **1**, formononetin; **2**, (2S)‐2’‐methoxykurarinone; **3**, kushenol E; **4**, kushenol F; **5**, (‐)‐maackiain; **6**, noranhydroicaritin; **7**, 4‐hydroxy‐3‐methoxy‐8,9‐methylenedioxypterocarpan; **8**, sophoraflavanone B.

Previously, we reported that treatment with total *S. flavescens* extracts to RAW‐Blue cells decreases *P. aeruginosa*‐mediated expression of IL‐1β by inhibiting NF‐κB activation [19]. To extend this observation, we obtained eight compounds derived from *S. flavescens* from KPEB (Supporting information) and examined the effect of each compound on NF‐κB activity in RAW‐Blue cells. The cells are mouse macrophage reporter cells, which are typically designed for the detection of NF‐κB activation through a SEAP reporter gene assay. As shown in Fig. 1A, six of the eight compounds (i.e., all except for **1** and **7**) suppressed activation of NF‐κB induced by *P. aeruginosa* strain PAO1, similar to the effects of the total extract (Ext). Next, we analyzed cytotoxicity of A549 cells by measuring the release of LDH. Compounds **1** and **5** as well as total Ext significantly decreased the release of LDH relative to the other compounds (Fig. 1B), suggesting that two compounds have low cytotoxicity. Thus, these data suggested that compound **5**‐mediated NF‐κB suppression is not caused by its cytotoxicity so that we select the compound **5** [(‐)‐maackiain; C_16_H_12_O_5_; MW 284.3] for further study.

### (‐)‐Maackiain amplifies the activation of caspase‐1 induced by nigericin

**Fig. 2:**
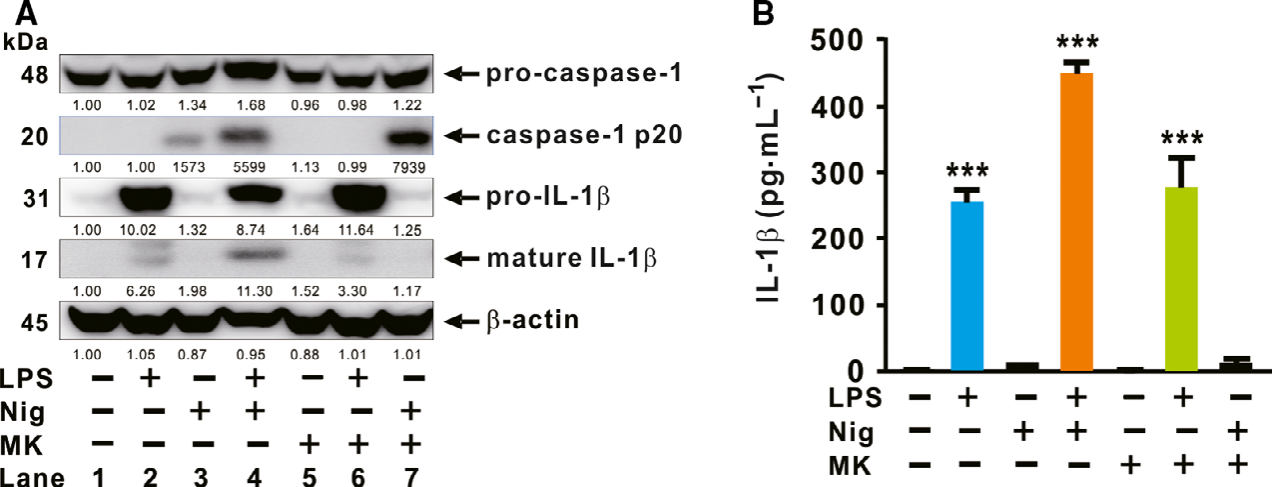
(‐)‐Maackiain amplifies the activation of caspase‐1 induced by nigericin. dTHP‐1 cells were seeded in 12‐well plates at a concentration of 7 × 10^5^ cell per mL, and the cells were primed by treatment with LPS and then treated sequentially with Nig and (‐)‐maackiain (MK). (A) Cleavages of pro‐caspase‐1 and pro‐IL‐1β were assessed by immunoblot analysis. Densitometry was shown below each immunoblot for quantitative analysis. (B) IL‐1β level was measured by cell ELISA. Immunoblot data in A are representative of three separate experiments. Data in B are expressed as means ± SD (*n* = 3). Student’s *t*‐test: ***P < 0.001 *vs*. no treatment.

Previously, it was reported that (‐)‐maackiain 3‐O‐glucoside as a (‐)‐maackiain analog inhibits LPS‐mediated expression of proinflammatory mediators, such as TNFα, IL‐6, and COX‐2, in macrophages probably through the inhibition of NF‐κB activation [10]. This led us to examine the effect of (‐)‐maackiain on the second activating signal, which is required for the maturation of IL‐1β through the activation of inflammasome/caspase‐1 [2]. To determine the effect, we first primed cells with LPS and then treated sequentially with nigericin and (‐)‐maackiain. Intriguingly, as shown in Fig. 2A, formation of the cleaved form (p20) of caspase‐1 was stimulated by treatment with nigericin and (‐)‐maackiain (Lane 7) to a much greater extent than treatment with nigericin alone (Lane 3). No cleavage was observed in response to treatment with (‐)‐maackiain alone (Lane 5). These imply that (‐)‐maackiain itself is not sufficient to initiate cleavage, but has an amplifying effect on the cleavage. Caspase‐1 cleavage was induced by the combined treatment with nigericin and (‐)‐maackiain (Lane 7) much more strongly than by treatment with two well‐known agonistic ligands, LPS and nigericin (Lane 4). However, treatment with LPS and (‐)‐maackiain (Lane 6) did not increase production of pro‐IL‐1β relative to treatment with LPS alone (Lane 2), suggesting that (‐)‐maackiain does not have an effect on the LPS‐mediated stimulation. This was further confirmed by measuring the release of mature IL‐1β, as shown in Fig. 2B. Taken together, these results demonstrate that (‐)‐maackiain plays a critical but discrete role in boosting the cleavage of caspase‐1 in response to nigericin.

### (‐)‐Maackiain‐activated caspase‐1 is biologically functional

**Fig. 3:**
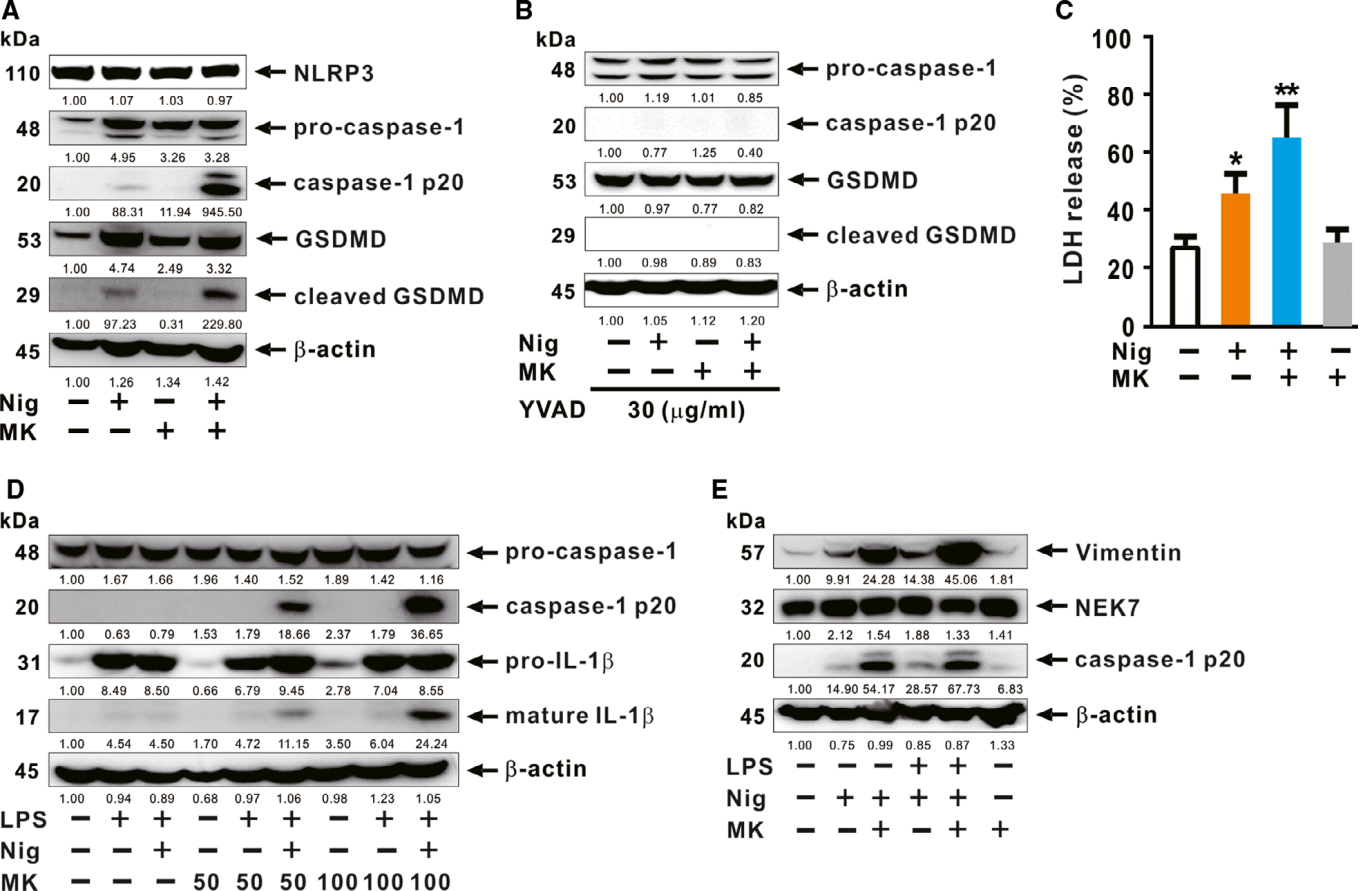
(‐)‐Maackiain‐activated caspase‐1 is biologically functional. dTHP‐1 cells were seeded in 12‐well plates at a concentration of 7 × 10^5^ cell per mL. (A‐C) The cells were treated sequentially with nigericin (Nig) and (‐)‐maackiain (MK). Cells were pretreated with the 30 µg·mL^−1^ of caspase‐1 inhibitor (YVAD) for 1 h (B). Immunoblot analysis (A, B) and LDH release assay (C) were performed. (D, E) The cells were primed by treatment with LPS and then treated sequentially with Nig and (‐)‐maackiain (MK). Immunoblot analysis was performed. Densitometry was shown below each immune blot for quantitative analysis. Immunoblot data are representative of three separate experiments. Data in C are expressed as means ± SD (*n* = 3). Student’s *t*‐test: *P < 0.05, **P < 0.01 *vs*. no treatment.

To obtain insight into the biological function of cleaved caspase‐1, we measured the level of cleaved GSDMD in macrophages. Cleavage of GSDMD is facilitated by the action of enzymatically active caspase‐1, which is crucial for canonical inflammasome activation. Then, the cleaved form of GSDMD induces pore formation in the plasma membrane, resulting in a distinctive form of programmed cell death known as pyroptosis, which facilitates the release of biologically active IL‐1β [3]. As shown in Fig. 3A, treatment with nigericin and (‐)‐maackiain promoted cleavage of pro‐caspase‐1 and GSDMD, which are assessed by immunoblot analysis. However, this treatment did not markedly alter production of NLRP3, which is under the control of NF‐κB, further supporting the idea that (‐)‐maackiain mainly stimulates caspase‐1 activation. No cleavage was observed in response to treatment with (‐)‐maackiain alone. Next, given that caspase‐4/5/11 required for noncanonical inflammasome activation triggers the cleavage of GSDMD, we sought to determine whether the (‐)‐maackiain promoted cleavage depends on caspase‐1 by pretreating with ac‐YVAD‐cmk (YVAD), a specific chemical inhibitor of the caspase‐1. As shown in Fig. 3B, the pretreatment clearly blocked the cleavage of GSDMD, indicating that (‐)‐maackiain‐mediated enhancement of GSDMD cleavage is specific to caspase‐1. The efficacy of inhibitor was confirmed by immunoblotting for cleaved form of caspase‐1. Consistent with the elevated level of cleaved GSDMD, cytotoxicity also gradually increased whereas treatment with (‐)‐maackiain alone did not cause cytotoxicity (Fig. 3C). Furthermore, treatment of LPS‐primed macrophages with nigericin and (‐)‐maackiain amplified not only the cleavage of pro‐caspase‐1, but also the production of mature IL‐1β, in a (‐)‐maackiain dose‐dependent manner (Fig. 3D). Next, we looked for mechanistic insights to address how (‐)‐maackiain might work to enhance the activation of caspase‐1. To achieve this, we analyzed the expression level of regulator proteins, such as vimentin and NEK7, which are required for the activation of NLRP3 inflammasome [21, 22]. As shown in Fig. 3E, (‐)‐maackiain treatment markedly increased the production of vimentin in response to nigericin but not NEK7, implying that (‐)‐maackiain‐mediated caspase‐1 activation could be attributed to the action of vimentin.

### (‐)‐Maackiain amplifies nigericin‐mediated caspase‐1 activation in MPL‐primed macrophages

**Fig. 4:**
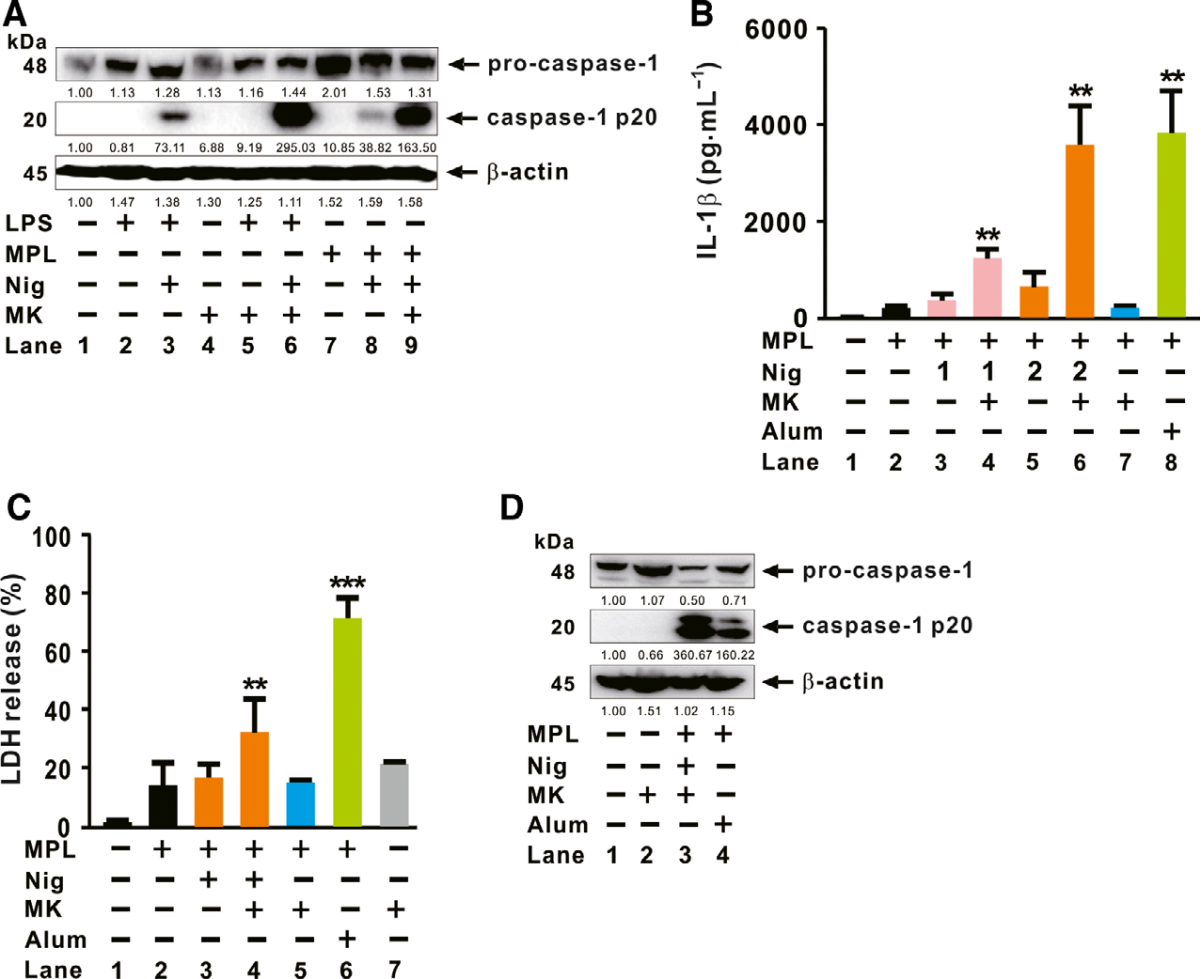
(‐)‐Maackiain amplifies nigericin‐mediated caspase‐1 activation in MPL‐primed macrophages. dTHP‐1 cells were seeded in 12‐well plates at a concentration of 7 × 10^5^ cell per mL, and the cells were primed with either LPS or MPL. (A) The cells were treated sequentially with Nig and (‐)‐maackiain (MK). Activation of caspase‐1 was assessed by immunoblot analysis. (B‐D) The cells were treated sequentially with Nig (1 or 2 µm for B; 2 µm for C and D) and either (‐)‐maackiain (MK) or alum. ELISA (B), LDH release assay (C), and immunoblot analysis (D) were performed. Densitometry was shown below each immune blot for quantitative analysis. Immunoblot data in A and D are representative of three separate experiments. Data in B and C are expressed as means ± SD (*n* = 3). Student’s *t*‐test: **P < 0.01, ***P < 0.001 *vs*. no treatment.

In contrast to LPS, MPL cannot activate the NLRP3 inflammasome and therefore fails to induce maturation of IL‐1β [23]. To determine whether (‐)‐maackiain can rescue the impaired activation of inflammasome in response to MPL, we compared the (‐)‐maackiain‐mediated cleavage of caspase‐1 between LPS‐ and MPL‐primed macrophages. As shown in Fig. 4A, consistent with previous reports, LPS priming (Lane 3) increased caspase‐1 cleavage to a greater extent than MPL priming (Lane 8). Furthermore, caspase‐1 activation in MPL‐primed macrophages was increased to a greater extent by supplement with (‐)‐maackiain in addition to nigericin (Lane 9) than treatment with nigericin (Lane 8). The increase shown at Lane 9 in MPL‐primed macrophages was even greater than that induced by treatment with nigericin in LPS‐primed macrophages (Lane 3; a well‐known agonistic ligand combination), implying that (‐)‐maackiain rescues impaired inflammasome activation by MPL. Next, because nigericin may cause cytotoxicity by decreasing the intracellular levels of ATP, GTP, and ADP through inhibition of cellular respiration [24], we asked whether the stimulatory effect of (‐)‐maackiain would still be observed upon treatment with lower concentrations of nigericin (1 or 2 µm). As shown in Fig. 4B, a clear increase in IL‐1β production was still observed upon treatment with lower levels of nigericin, providing further proof of the compound’s ability to stimulate inflammasome activation.

To boost the adjuvant potential of MPL, GlaxoSmithKline Biologicals developed a combination adjuvant designated AS04, which contains alum [25, 26]. Given that MPL cannot activate NLRP3 inflammasome, the addition of alum complements the potential of MPL, resulting in activation of the NLRP3 inflammasome and in subsequent production of mature IL‐1β [27, 28]. We sought to determine whether (‐)‐maackiain‐mediated activation of the inflammasome is comparable to the effect mediated by alum in MPL‐primed macrophages. As shown in Fig. 4B, in MPL‐primed macrophages, IL‐1β release was stimulated by treatment with nigericin and (‐)‐maackiain (Lane 6), similar to the effect of treatment with alum (Lane 8), indicating that (‐)‐maackiain had a comparable stimulatory effect on the release of IL‐1β. Consistent with Fig. 4A (Lanes 8 and 9), treatment of MPL‐primed macrophages with (‐)‐maackiain in addition to nigericin (Lane 6 in Fig. 4B) stimulated release of IL‐1β to a greater extent than treatment with nigericin (Lane 5 in Fig. 4B). In addition, treatment of MPL‐primed macrophages with (‐)‐maackiain (Lane 7 in Fig. 4B) did not increase IL‐1β release relative to MPL priming alone (Lane 2 in Fig. 4B), again verifying the effect of (‐)‐maackiain on inflammasome activation, but not on the state created by the priming signal (i.e., MPL treatment). Then, we compared the cytotoxicity of (‐)‐maackiain and alum. As shown in Fig. 4C, cytotoxicity was lower in MPL‐primed macrophages treated with nigericin (2 µm) and (‐)‐maackiain (Lane 4) than in cells treated with alum (Lane 6), although cytotoxicity in Lane 4 was slightly higher than that induced by the treatment of MPL‐primed macrophages with either nigericin (Lane 3) or (‐)‐maackiain (Lane 5). Finally, we analyzed the cleavage of caspase‐1 to understand whether (‐)‐maackiain treatment stimulates more efficiently inflammasome activation than alum. As shown in Fig. 4D, treatment of MPL‐primed macrophages with nigericin and (‐)‐maackiain (Lane 3) stimulated the cleavage of caspase‐1 to a greater extent than treatment with alum (Lane 4). Taken together, these findings demonstrate that, in MPL‐primed macrophages, (‐)‐maackiain is less cytotoxic than alum but more efficiently stimulates activation of the inflammasome.

## Discussion

In this study, we demonstrated that *S. flavescens*‐derived compound, (‐)‐maackiain, has an activating effect on inflammasome, which is proven by the increase in caspase‐1 cleavage once treated with nigericin, and this can lead to enhanced production of IL‐1β in either LPS‐ or MPL‐primed cells. (‐)‐Maackiain does not have an effect on stimulation by the first priming signal, LPS treatment (Lane 6 in Fig. 2A), but can intensify the state promoted by nigericin treatment (Lane 7 in Figs 2A and 3A), which is used as the second activating signal required for stimulation of inflammasome. (‐)‐Maackiain has inhibiting effects on the activation of NF‐κB based on a reporter assay with RAW‐Blue cells (Fig. 1A) so that we first primed cells with LPS or MPL and then treated sequentially with nigericin and (‐)‐maackiain to determine its role on the stimulation of inflammasome. In addition, we switched the target cell to THP‐1 because RAW‐Blue cells do not express ASC [29] and A549 cells do not produce caspase‐1 [30]. Nigericin treatment alone slightly promoted the cleavage of caspase‐1 (Figs 2A and 3A), suggesting that the nigericin‐mediated cleavage is to some extent independent of a priming signal such as LPS. This is in agreement with a previous report that ATP‐induced release of mature IL‐18 is independent of new protein synthesis in response to LPS [31]. Thus, (‐)‐maackiain plays a role in boosting the cleavage of caspase‐1 in response to nigericin. In addition, we clearly observed the increase of IL‐1β production in response to LPS alone as shown in Fig. 2B. Unlike classical inflammasome promoted by priming and activating signals, this could be mediated by alternative inflammasome, which promotes the secretion of IL‐1β in response to LPS alone [32]. These imply that (‐)‐maackiain‐mediated stimulation depends on the action of nigericin but not that of LPS. Notably, the upregulation of caspase‐1 cleavage depicted in Lane 7 in Fig. 2A was only observed in macrophages cultured in the presence of serum, suggesting that (‐)‐maackiain‐mediated activation of caspase‐1 is dependent on the action of additional factors present in serum.

Like ATP, nigericin stimulates the P2X7 receptor, thereby decreasing intracellular potassium levels and activating the NLRP3‐associated inflammasome via the classical pathway [33, 34]. Here, (‐)‐maackiain is actively involved in the activation of inflammasome by contributing to the action of nigericin on the classical inflammasome pathway after the priming step. In the course of study, we examined the level of regulator proteins, NEK7 and vimentin, which have shown their roles as essential mediators for the activation of NLRP3 inflammasome [21, 22], to understand mechanisms underlying the process of enhancement. As a result, we found that (‐)‐maackiain increased the production of intermediate filaments, vimentin (Fig. 3E), which acts as a scaffold for the assembly of NLRP3 inflammasome complex by promoting interaction with caspase‐1 [22]. However, the enhancement of vimentin expression was not observed by treatment with (‐)‐maackiain alone, further supporting the effect of (‐)‐maackiain in promoting the formation of inflammasome complex to activate caspase‐1 in response to nigericin. In addition, treatment with nigericin and (‐)‐maackiain more strongly increased the production of vimentin than with LPS and nigericin, leading to higher cleavage of caspase‐1.

Monophosphoryl lipid A, a detoxified component of LPS, has been used as an adjuvant to increase vaccine efficacy [35], and it activates key innate immune pathways through the action of TLR4 [36]. However, unlike LPS‐mediated induction of TLR4 activation through the TRIF and MyD88 pathways, MPL leads to activation only through TRIF, resulting in lower levels of inflammatory responses [37]. In addition, MPL cannot activate the NLRP3 inflammasome in contrast to LPS, and NLRP3 inflammasome plays a critical role in limiting lung damage resulting from influenza infections [38, 39]. The resultant production of IL‐1β stimulates acute inflammatory responses that play critical roles in defending against influenza infections [4]. Therefore, MPL fails to induce maturation of IL‐1β [23] and consequently fails to achieve efficient vaccination against influenza infection [38, 39, 40]. This implies that inflammasome signaling represents a promising target for improving MPL‐mediated vaccine efficacy. Especially, elderly mice infected with influenza produce less amounts of active IL‐1β even though they have normal levels of pro‐IL‐1β [7]. Thus, the potent inflammasome‐activating compound like (‐)‐maackiain could be useful clinically as an immune‐stimulating agent and supplemental influenza vaccine adjuvant to further increase caspase‐1‐augmented IL‐1β production.

## Conflict of interest

The authors declare no conflict of interest.

## Author contributions

JWH and JHL planned the experiments. JWH, JHL, EJ, and HWR performed the experiments. HWR, SRO, and KSA analyzed the data. HSJ and UHH supervised the study. JWH and JHL wrote the original draft of the manuscript. UHH wrote, reviewed, and edited the manuscript.

## Supporting information


**Fig. S1‐1**. MPLC fractionation of *Sophora flavescens* extract.
**Fig. S1‐2**. UPLC‐PDA‐QTof‐MS of fractions of *Sophora flavescens* extract.
**Fig. S1‐3**. UPLC‐PDA‐QTof‐MS of fractions of *Sophora flavescens* extract.
**Fig. S2‐1**. UV, MS/MS, and MS data of formononetin **1**.
**Fig. S2‐2**. UV, MS/MS, and MS data of (2S)‐2'‐methoxy kurarinone **2**.
**Fig. S2‐3**. UV, MS/MS, and MS data of kushenol E **3**.
**Fig. S2‐4**. UV, MS/MS, and MS data of kushenol F **4**.
**Fig. S2‐5**. UV, MS/MS, and MS data of (‐)‐maackiain **5**.
**Fig. S2‐6**. UV, MS/MS, and MS data of noranhydroicaritin **6**.
**Fig. S2‐7**. UV, MS/MS, and MS data of (‐)‐4‐hydroxy‐3‐methoxy‐8,9‐methylenedioxypterocarpan **7**.
**Fig. S2‐8**. UV, MS/MS, and MS data of sophoraflavanone B **8**.
**Fig. S3‐1**. ^1^H‐NMR spectrum of formononetin **1** (400 MHz, DMSO‐*d_6_*).
**Fig. S3‐2**. ^13^C‐NMR spectrum of formononetin **1** (100 MHz, DMSO‐*d_6_*).
**Fig. S3‐3**. ^1^H‐NMR spectrum of (2S)‐2'‐methoxy kurarinone **2** (400 MHz, DMSO‐*d_6_*).
**Fig. S3‐4**. ^13^C‐NMR spectrum of (2S)‐2'‐methoxy kurarinone **2** (100 MHz, DMSO‐*d_6_*).
**Fig. S3‐5**. ^1^H‐NMR spectrum of kushenol E **3** (400 MHz, DMSO‐*d_6_*).
**Fig. S3‐6**. ^13^C‐NMR spectrum of kushenol E **3** (100 MHz, DMSO‐*d_6_*).
**Fig. S3‐7**. ^1^H‐NMR spectrum of kushenol F **4** (400 MHz, DMSO‐*d_6_*).
**Fig. S3‐8**. ^13^C‐NMR spectrum of kushenol F **4** (100 MHz, DMSO‐*d_6_*).
**Fig. S3‐9**. ^1^H‐NMR spectrum of (‐)‐maackiain **5** (400 MHz, DMSO‐*d_6_*).
**Fig. S3‐10**. ^13^C‐NMR spectrum of (‐)‐maackiain **5** (100 MHz, DMSO‐*d_6_*).
**Fig. S3‐11**. ^1^H‐NMR spectrum of noranhydroicaritin **6** (400 MHz, DMSO‐*d_6_*).
**Fig. S3‐12**. ^13^C‐NMR spectrum of noranhydroicaritin **6** (100 MHz, DMSO‐*d_6_*).
**Fig. S3‐13**. ^1^H‐NMR spectrum of (‐)‐4‐hydroxy‐3‐methoxy‐8,9‐methylenedioxypterocarpan **7** (400 MHz, DMSO‐*d_6_*).
**Fig. S3‐14**. ^13^C‐NMR spectrum of (‐)‐4‐hydroxy‐3‐methoxy‐8,9‐methylenedioxypterocarpan **7** (100 MHz, DMSO‐*d_6_*).
**Fig. S3‐15**. ^1^H‐NMR spectrum of sophoraflavanone B **8** (400 MHz, DMSO‐*d_6_*).
**Fig. S3‐16**. ^13^C‐NMR spectrum of sophoraflavanone B **8** (100 MHz, DMSO‐*d_6_*).
