# Supplementary material for: Maackiain, a compound derived from Sophora flavescens, increases IL‐1β production by amplifying nigericin‐mediated inflammasome activation
Source: FEBS Open Bio. 2020 Jun 26;10(8):1482–91. doi: 10.1002/2211-5463.12899 (PMC7396426; doi:10.1002/2211-5463.12899)
Supplement: Supplementary file 1 — Fig. S1‐1. MPLC fractionation of Sophora flavescens extract. Fig. S1‐2. UPLC‐PDA‐QTof‐MS of fractions of Sophora flavescens extract. Fig. S1‐3. UPLC‐PDA‐QTof‐MS of fractions of Sophora flavescens extract. Fig. S2‐1. UV, MS/MS, and MS data of formononetin 1. Fig. S2‐2. UV, MS/MS, and MS data of (2S)‐2'‐methoxy kurarinone 2. Fig. S2‐3. UV, MS/MS, and MS data of kushenol E 3. Fig. S2‐4. UV, MS/MS, and MS data of kushenol F 4. Fig. S2‐5. UV, MS/MS, and MS data of (‐)‐maackiain 5. Fig. S2‐6. UV, MS/MS, and MS data of noranhydroicaritin 6. Fig. S2‐7. UV, MS/MS, and MS data of (‐)‐4‐hydroxy‐3‐methoxy‐8,9‐methylenedioxypterocarpan 7. Fig. S2‐8. UV, MS/MS, and MS data of sophoraflavanone B 8. Fig. S3‐1. 1H‐NMR spectrum of formononetin 1 (400 MHz, DMSO‐d6). Fig. S3‐2. 13C‐NMR spectrum of formononetin 1 (100 MHz, DMSO‐d6). Fig. S3‐3. 1H‐NMR spectrum of (2S)‐2'‐methoxy kurarinone 2 (400 MHz, DMSO‐d6). Fig. S3‐4. 13C‐NMR spectrum of (2S)‐2'‐methoxy kurarinone 2 (100 MHz, DMSO‐d6). Fig. S3‐5. 1H‐NMR spectrum of kushenol E 3 (400 MHz, DMSO‐d6). Fig. S3‐6. 13C‐NMR spectrum of kushenol E 3 (100 MHz, DMSO‐d6). Fig. S3‐7. 1H‐NMR spectrum of kushenol F 4 (400 MHz, DMSO‐d6). Fig. S3‐8. 13C‐NMR spectrum of kushenol F 4 (100 MHz, DMSO‐d6). Fig. S3‐9. 1H‐NMR spectrum of (‐)‐maackiain 5 (400 MHz, DMSO‐d6). Fig. S3‐10. 13C‐NMR spectrum of (‐)‐maackiain 5 (100 MHz, DMSO‐d6). Fig. S3‐11. 1H‐NMR spectrum of noranhydroicaritin 6 (400 MHz, DMSO‐d6). Fig. S3‐12. 13C‐NMR spectrum of noranhydroicaritin 6 (100 MHz, DMSO‐d6). Fig. S3‐13. 1H‐NMR spectrum of (‐)‐4‐hydroxy‐3‐methoxy‐8,9‐methylenedioxypterocarpan 7 (400 MHz, DMSO‐d6). Fig. S3‐14. 13C‐NMR spectrum of (‐)‐4‐hydroxy‐3‐methoxy‐8,9‐methylenedioxypterocarpan 7 (100 MHz, DMSO‐d6). Fig. S3‐15. 1H‐NMR spectrum of sophoraflavanone B 8 (400 MHz, DMSO‐d6). Fig. S3‐16. 13C‐NMR spectrum of sophoraflavanone B 8 (100 MHz, DMSO‐d6). [file FEB4-10-1482-s001.doc]

**SUPPLEMENTARY DATA**

**Instruments**

NMR spectra were recorded on a Bruker AM500 instrument (^1^H NMR at 500 MHz, ^13^C NMR at 125 MHz, Bruker, Karlsruhe, Germany) and tetramethylsilane was used as the internal standard. Chemical shifts are reported in ppm (parts per million), and coupling constants (*J*) in Hz. Ultra-performance liquid chromatography (UPLC) was performed using an ACQUITY UPLC™ system (Waters Corporation, Milford, MA, USA) equipped with a binary solvent delivery manager, a photodiode array (PDA). HRESIMS was performed on a Waters Q-TOF Premier^TM^ mass spectrometer equipped with an electrospray interface (Waters Corporation, Milford, MA, USA). Separations were conducted on an Armen Spot prep II 250 medium pressure liquid chromatograph (MPLC) and on a PLC2020 prep–high performance liquid chromatograph (prep-HPLC) (Gilson, Inc. Middleton, WI, USA) using an appropriately size reversed-phase silica gel column purchased from Waters. All solvents used for column chromatography were of analytical grade (SK Chemicals Co. Ltd., Seongnam, Korea), and all solvents used for HPLC were of HPLC grade (SK Chemicals Co., Ltd.). NMR solvents were purchased from Cambridge Isotope Laboratory Inc. (Andover, MA, USA).

**
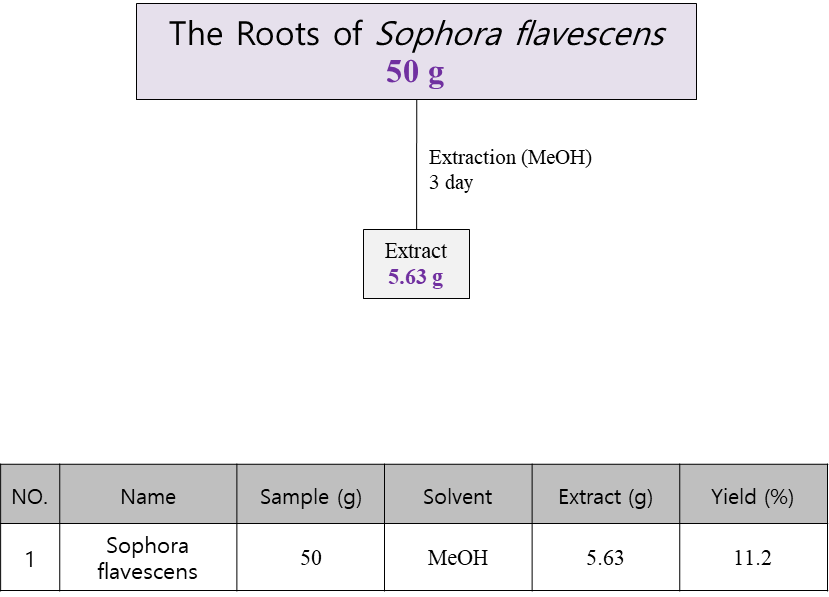
**

**
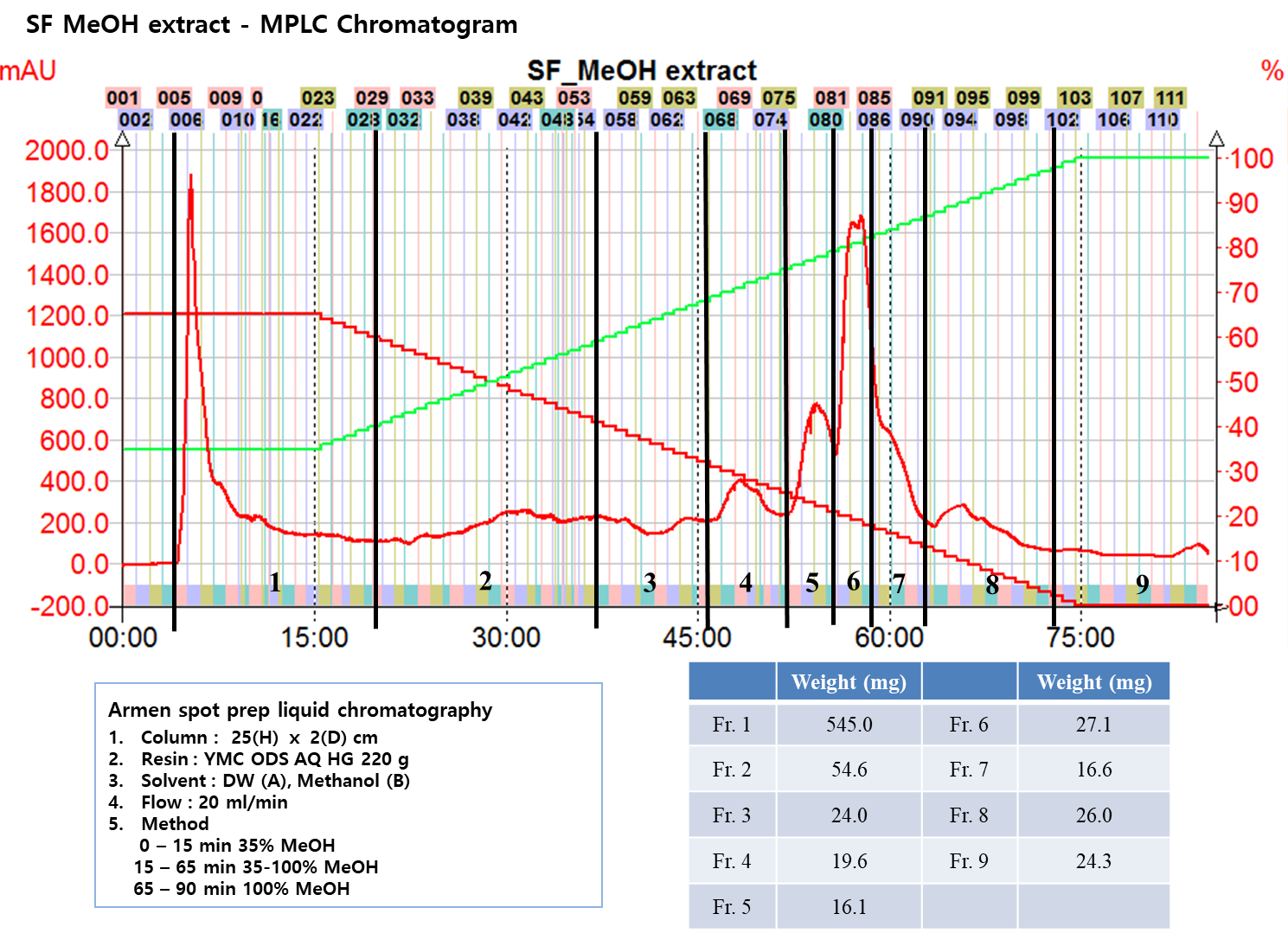
**

**Figure S1-1.** MPLC fractionation of *Sophora flavescens* extract.


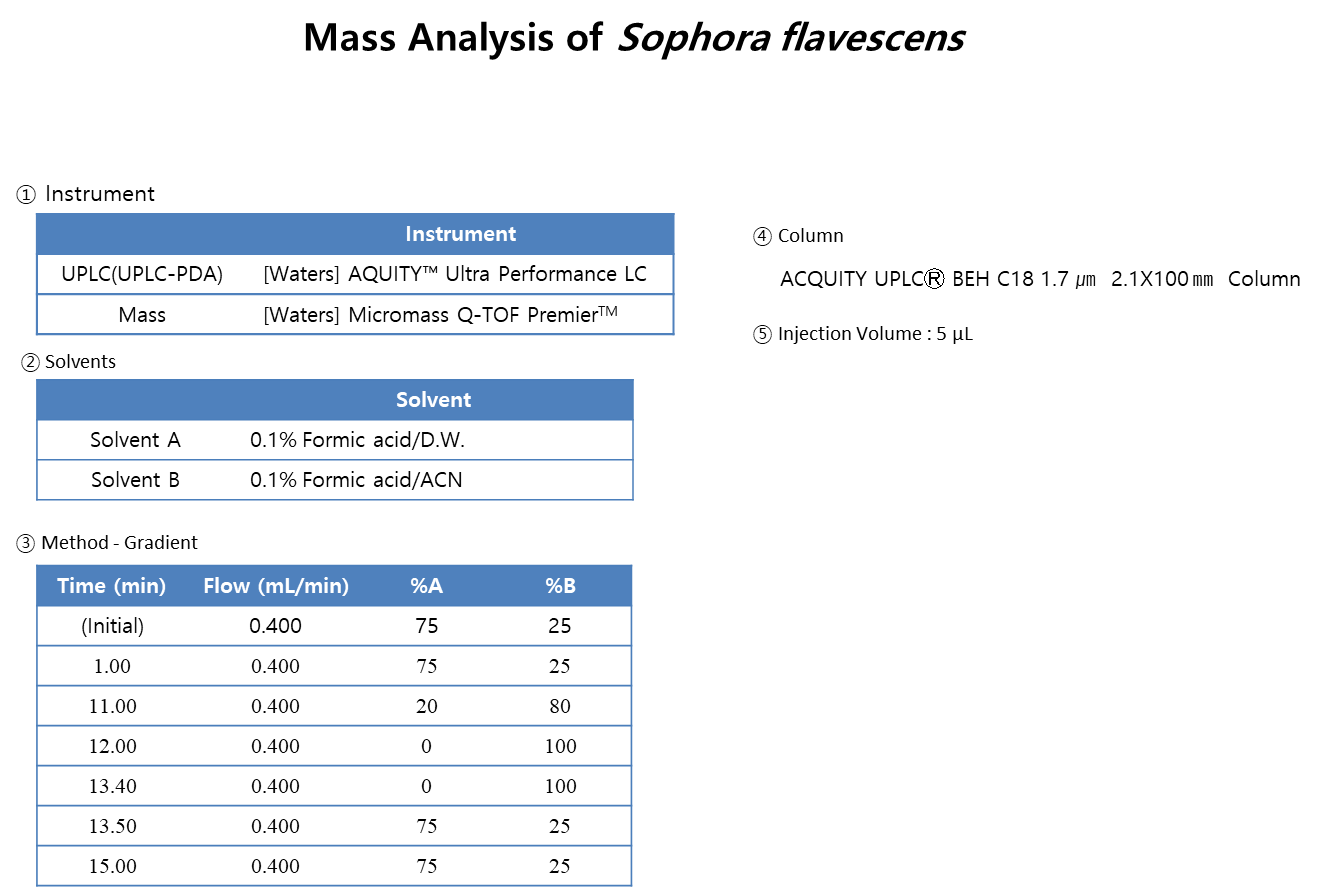


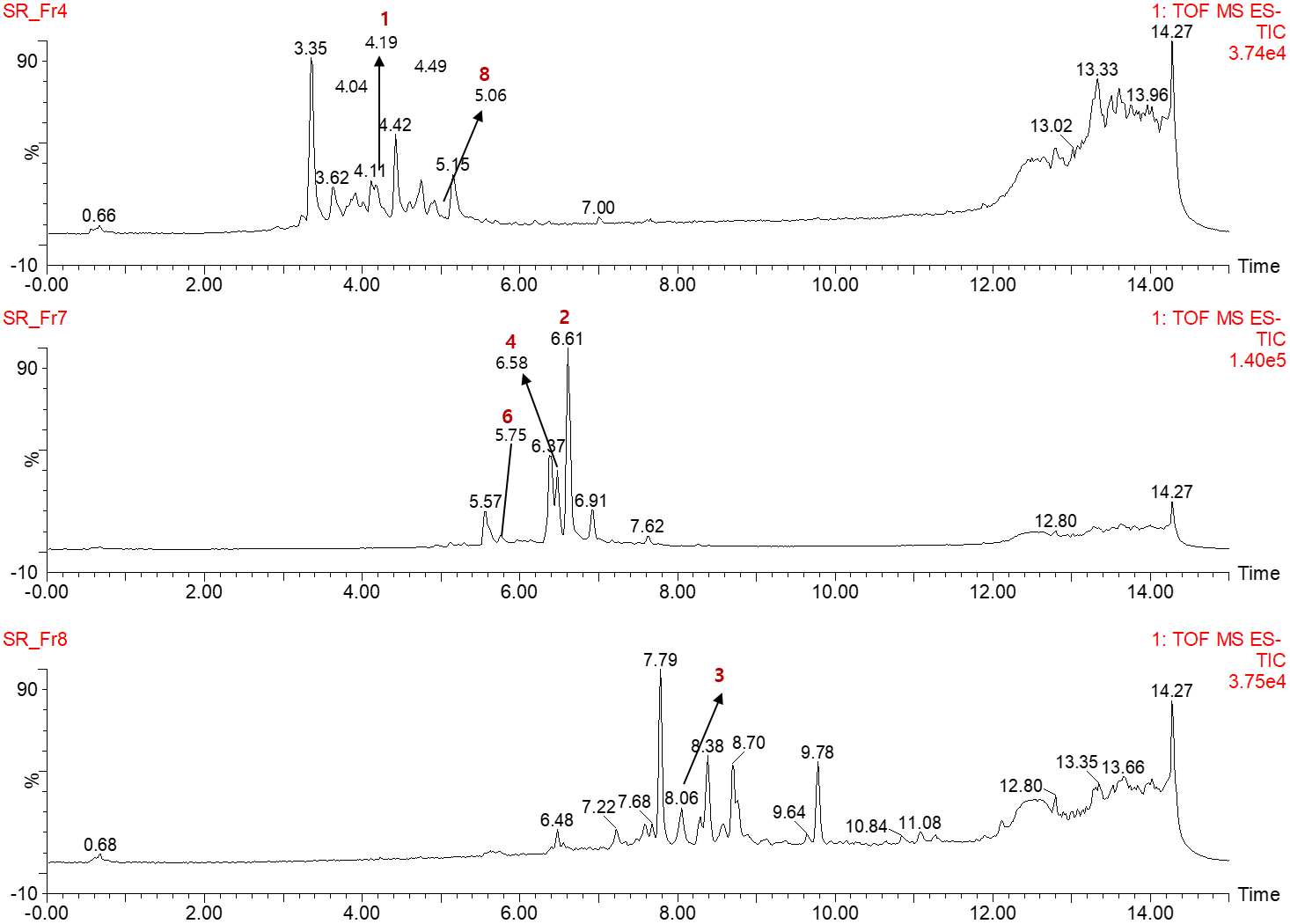


**Figure S1-2.** UPLC-PDA-QTof-MS of fractions of *Sophora flavescens* extract.

**
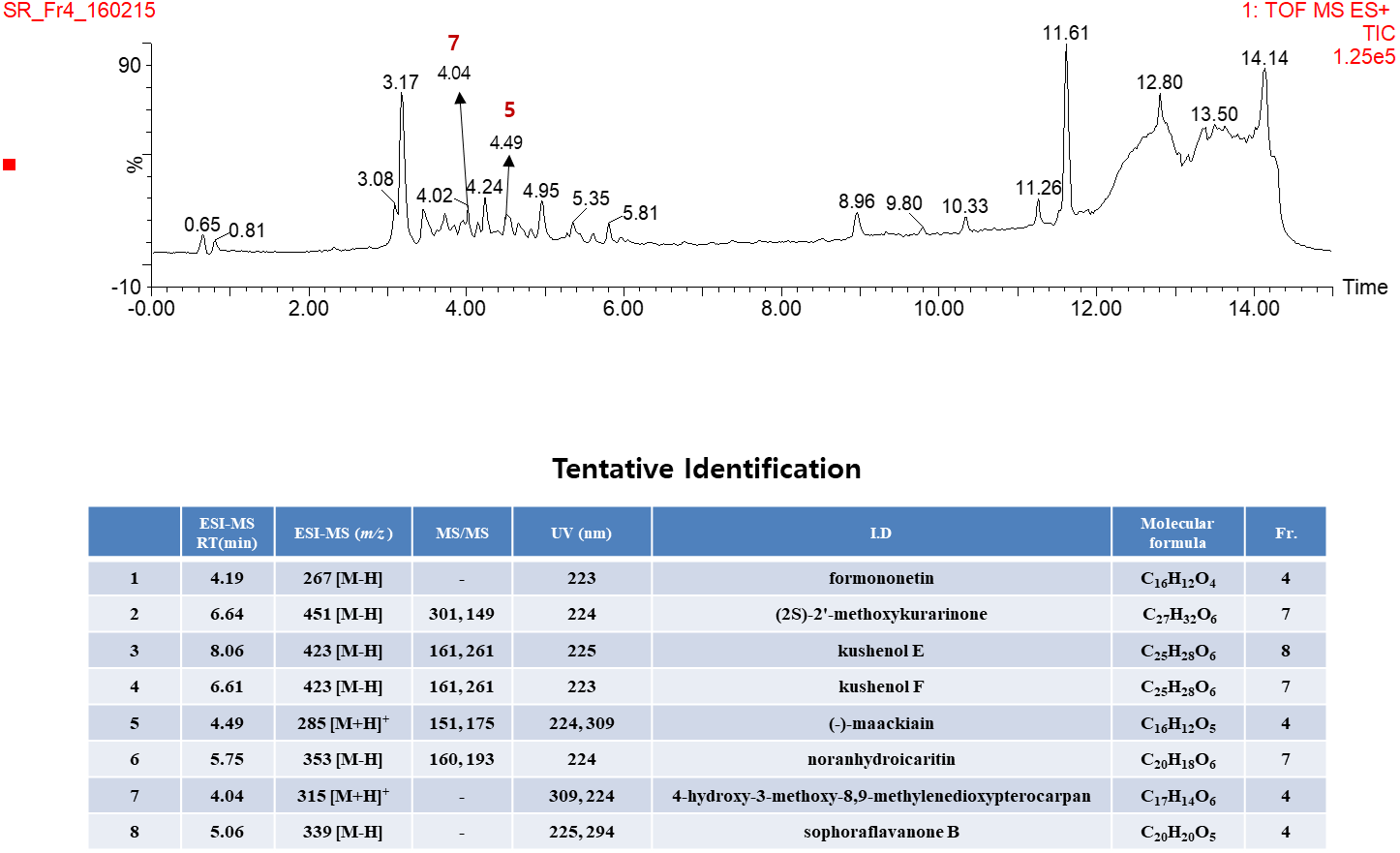
**

**Figure S1-3.** UPLC-PDA-QTof-MS of fractions of *Sophora flavescens* extract.


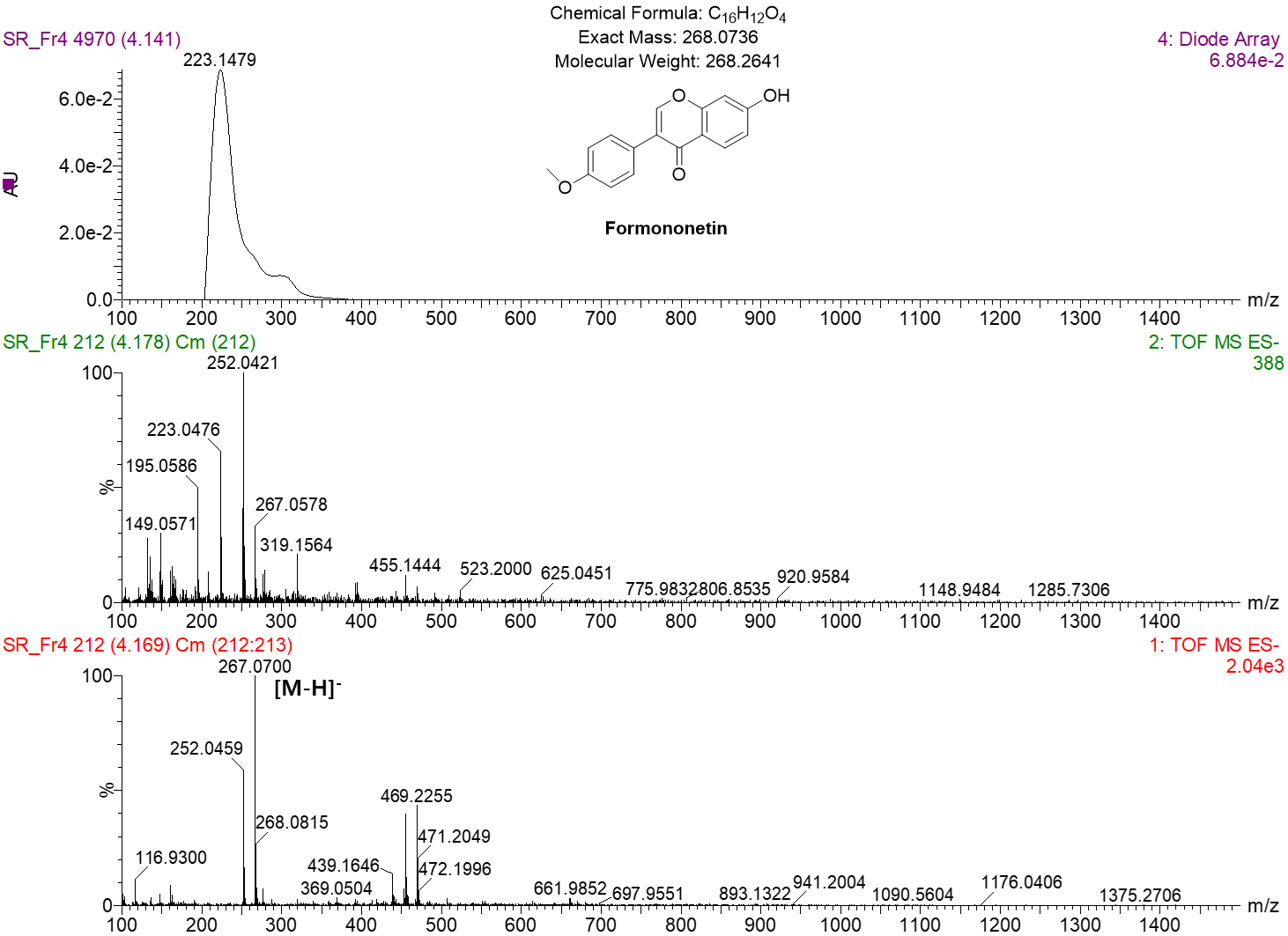


**Figure S2-1.** UV, MS/MS, and MS data of formononetin **1**.


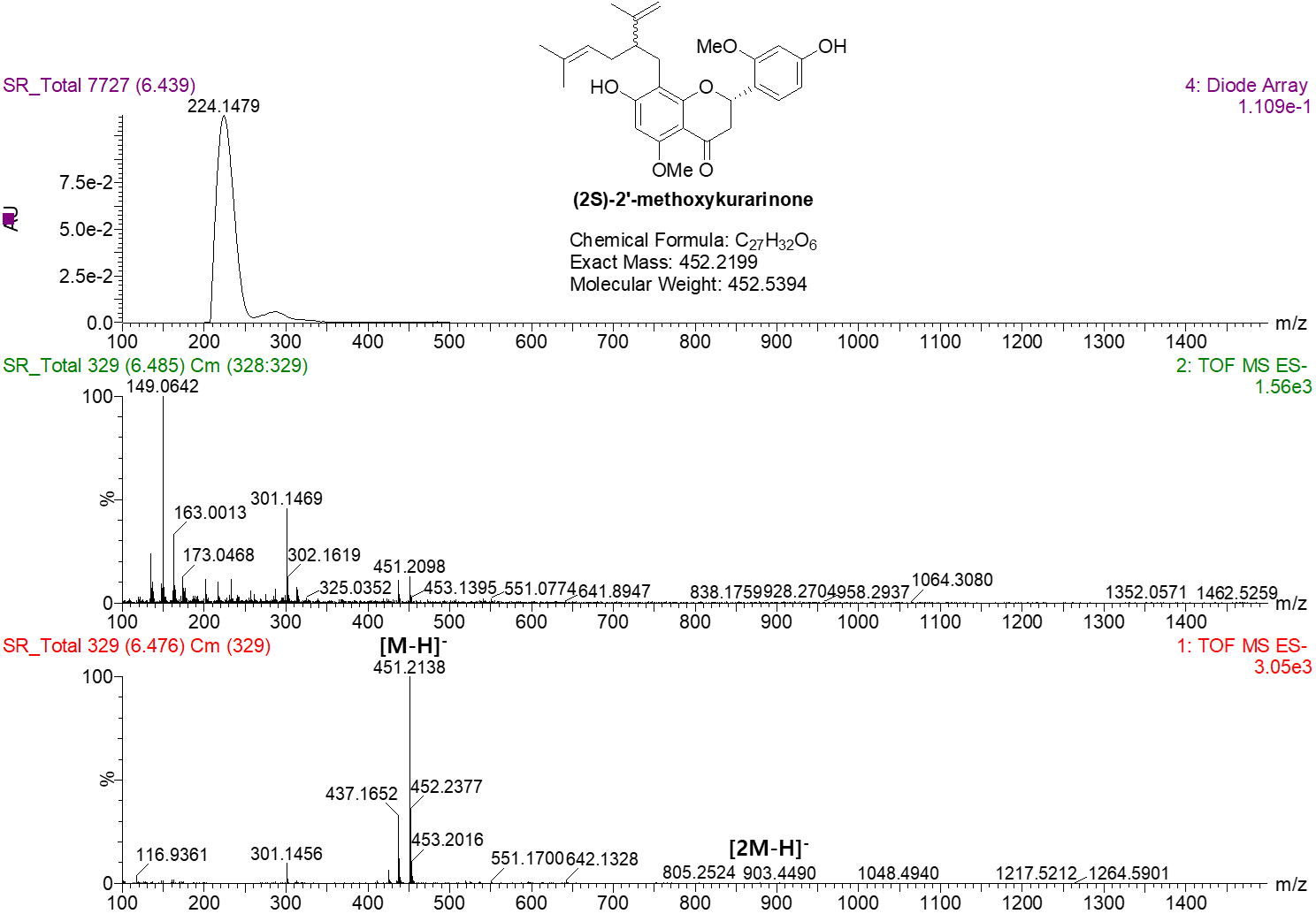


**Figure S2-2.** UV, MS/MS, and MS data of (2S)-2'-methoxy kurarinone **2**.


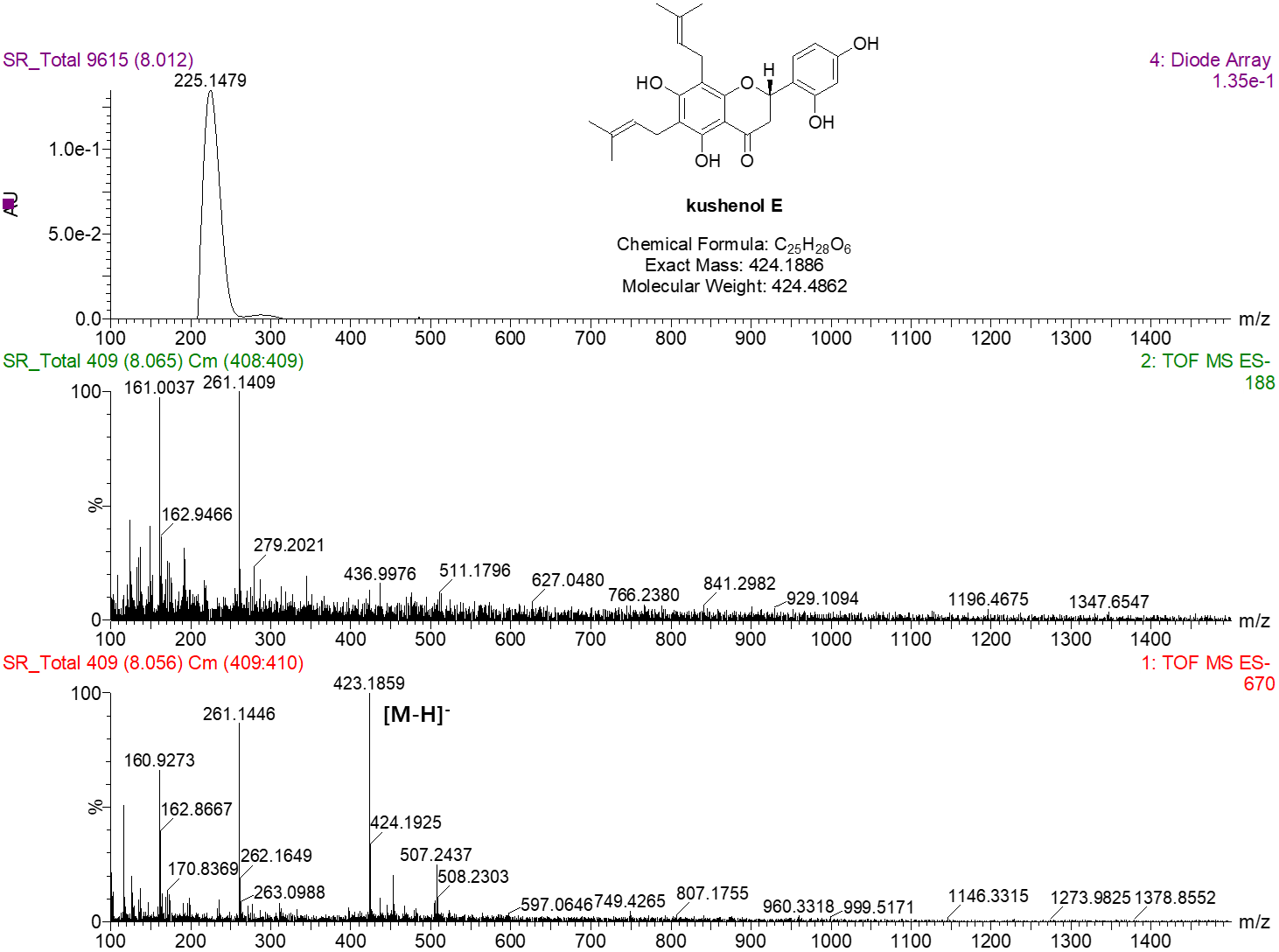


**Figure S2-3.** UV, MS/MS, and MS data of kushenol E **3**.


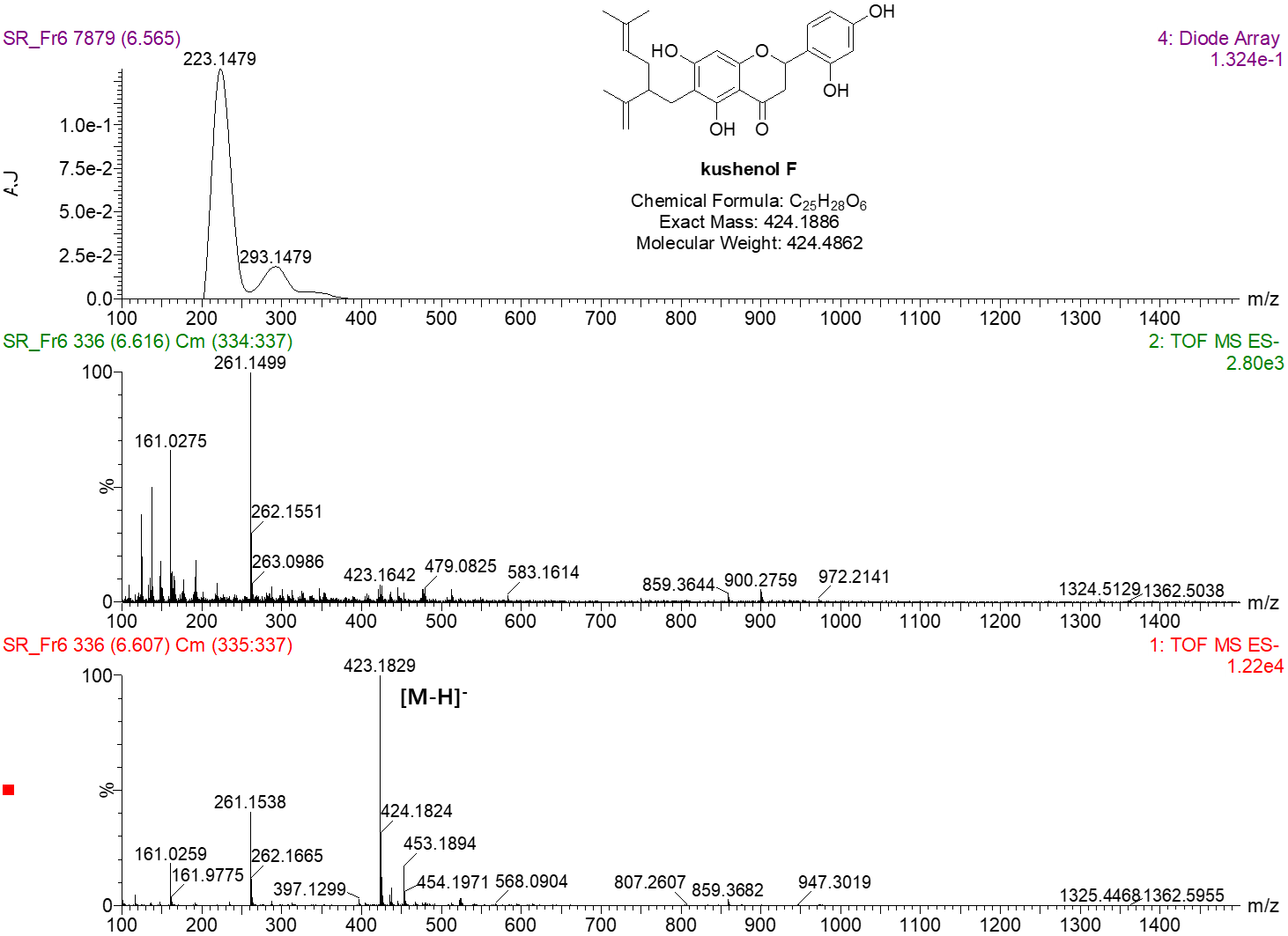


**Figure S2-4.** UV, MS/MS, and MS data of kushenol F **4**.


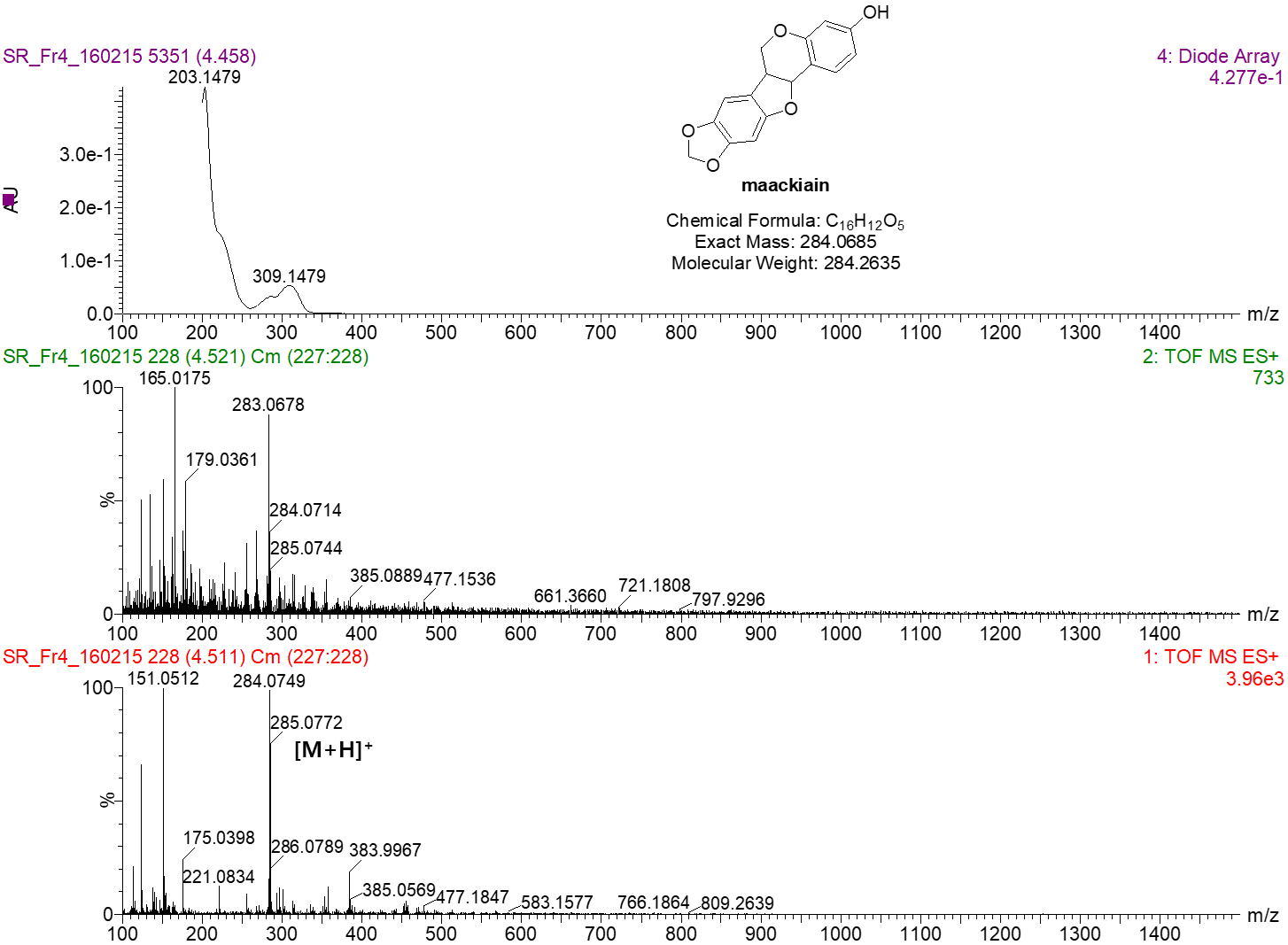


**Figure S2-5.** UV, MS/MS, and MS data of (-)-maackiain **5**.


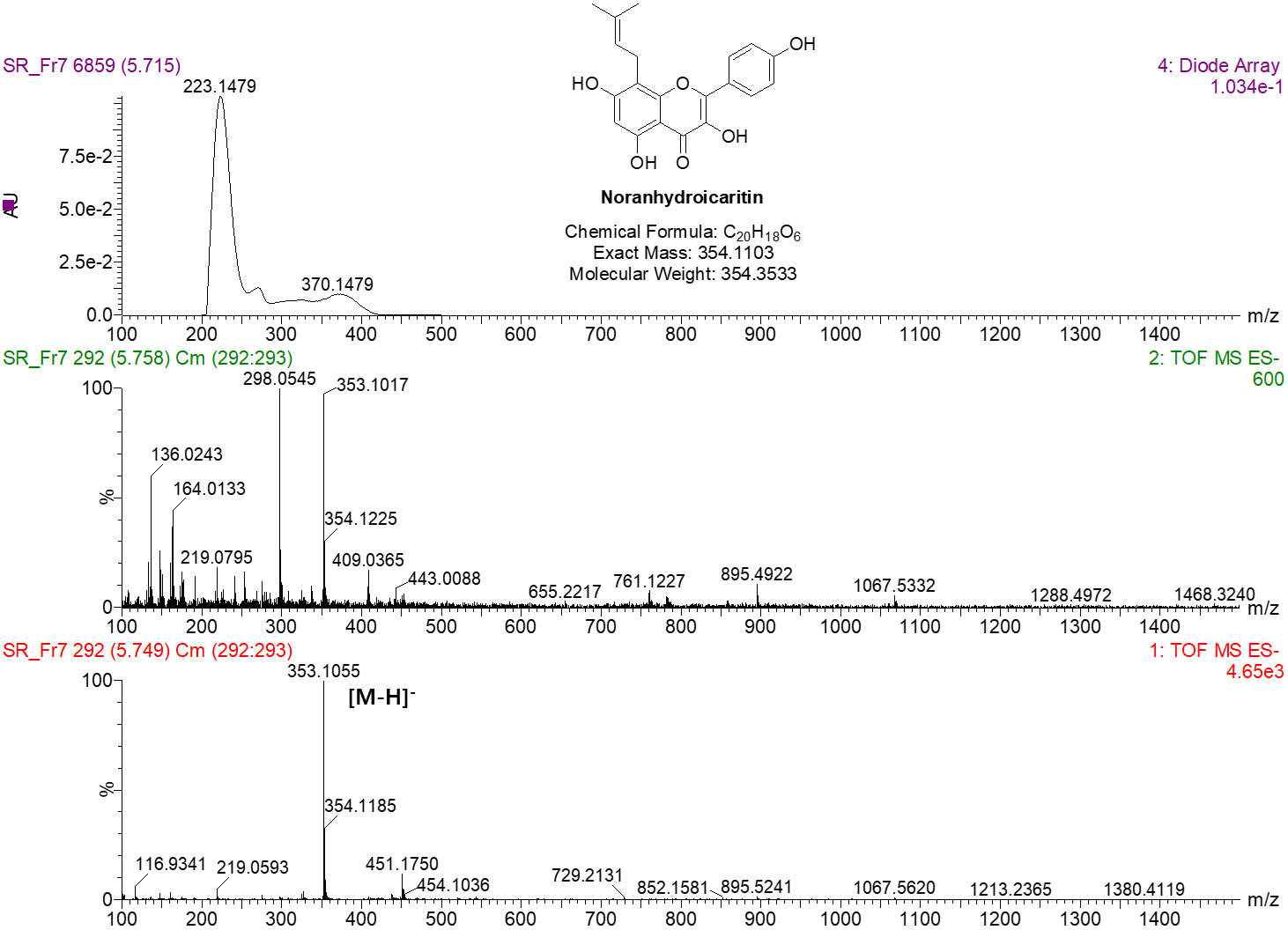


**Figure S2-6.** UV, MS/MS, and MS data of noranhydroicaritin **6**.


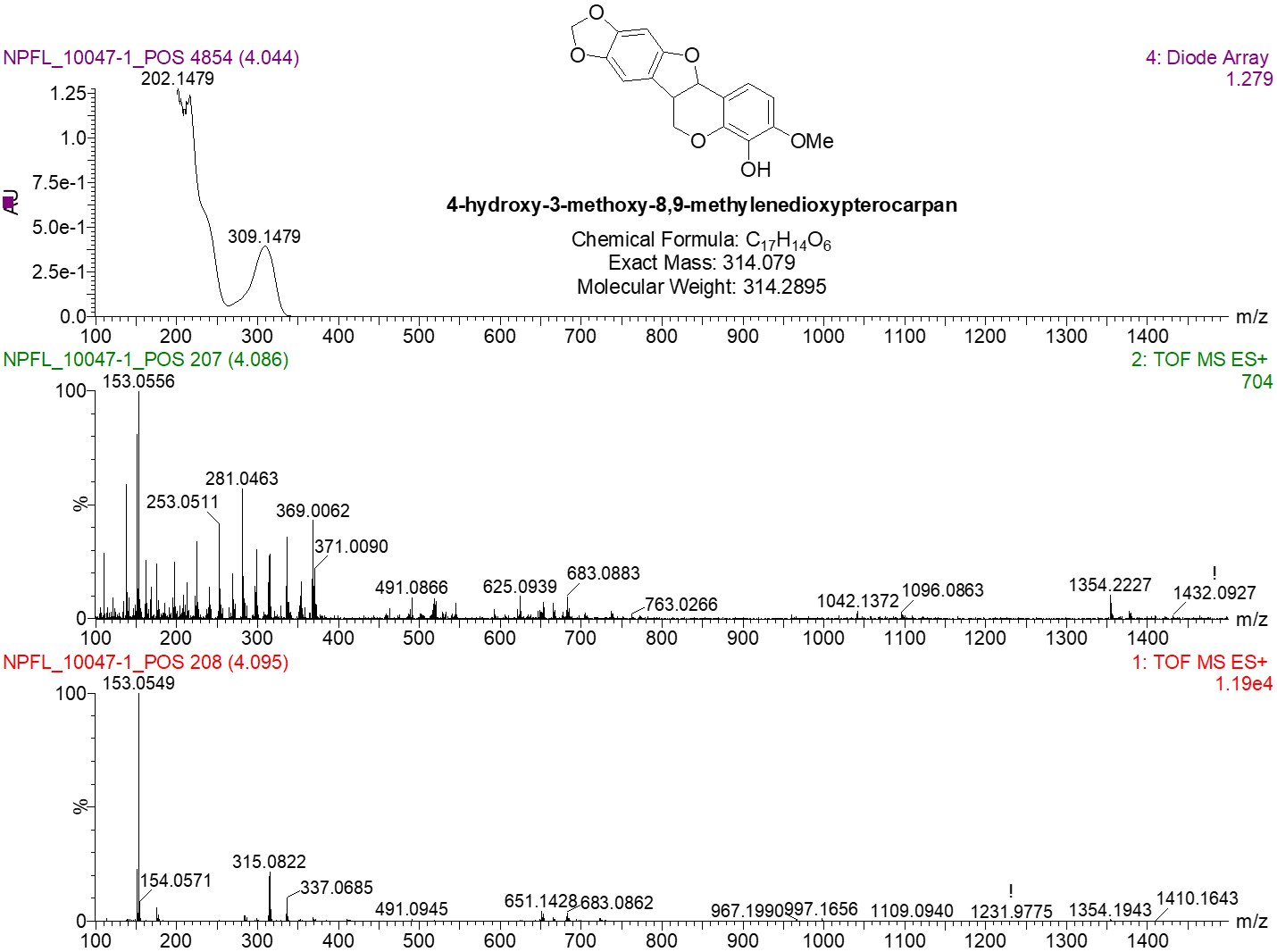


**Figure S2-7.** UV, MS/MS, and MS data of (-)-4-hydroxy-3-methoxy-8,9-methylenedioxypterocarpan **7**.


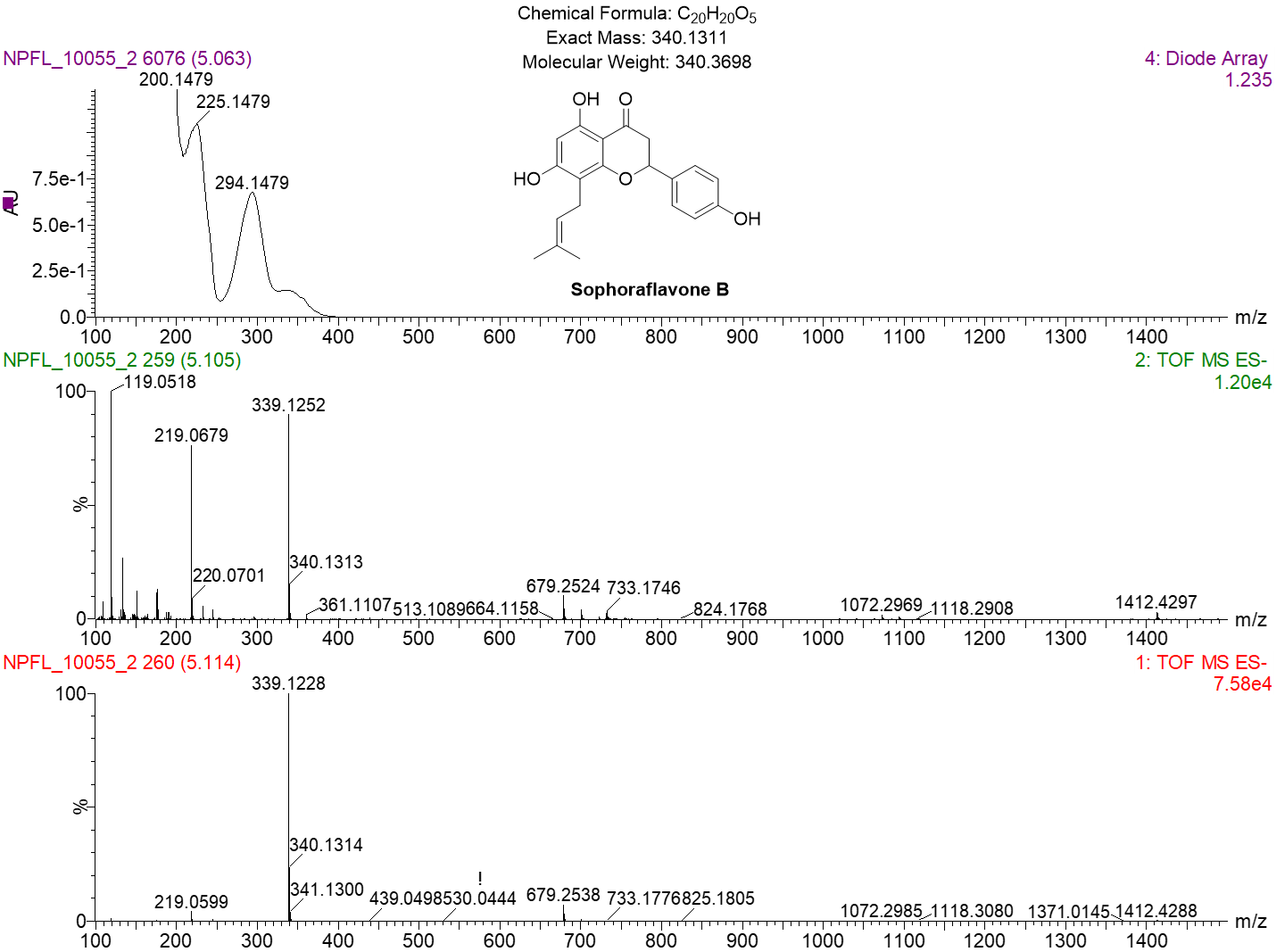


**Figure S2-8.** UV, MS/MS, and MS data of sophoraflavanone B **8**.

Formononetin (**1**): m.p. 255-257 °C; HRESIMS *m/z* 267.0660 [M‒H]^‒^ (calcd for C_16_H_11_O_4_, 267.0657); ^1^H NMR (300 MHz, DMSO-*d_6_*) *δ* 3.87 (s, 4'-OCH_3_), 6.86 (1H, d, *J* = 2.3 Hz, H-8), 7.05 (2H, d, *J* = 8.9 Hz, H-3',5'), 7.14 (1H, d, *J* = 8.8 Hz, H-6), 7.50 (2H, d, *J* = 8.9 Hz, H-2',6'), 7.98 (1H, d, *J* = 8.8 Hz, H-5), 8.32 (1H, s, H-2); ^13^C NMR (75 MHz, DMSO-*d_6_*) *δ* 55.1 (OCH_3_), 102.1 (C-8), 113.6 (C-5'), 113.6 (C-3',5'), 115.2 (C-6), 116.5 (C-10), 123.2 (C-1'), 124.2 (C-3), 127.3 (C-5), 130.0 (C-2 ',6'), 153.2 (C-2), 157.4 (C-9), 158.9 (C-4'), 162.6 (C-7), 174.5 (C-4).

(2S)-2'-Methoxy kurarinone (**2**): yellow powder; m.p. 112-115 °C; [α]_D_^20^ -135.4° (c 2.0, MeOH); HRESIMS *m/z* 451.2138 [M‒H]^‒^ (calcd for C_27_H_31_O_6_, 451.2121); ^1^H NMR (500 MHz, acetone-*d_6_*) *δ* 1.45 (3H, s, H-6''), 1.56 (3H, s, H-7''), 1.63 (3H, s, H-9''), 2.00 (2H, m, H-3''), 2.49 (1H, m, H-2''), 2.62 (2H, m, H-1''), 2.59 (1H, dd, *J* = 6.7, 2.9 Hz, H-3), 2.87 (1H, dd, *J* = 16.7, 3.2 Hz, H-3), 3.79 (3H, s, H-11''), 3.80 (3H, s, H-12''), 4.57 (2H, m, H-10''), 4.96 (1H, m, H-4''), 5.54 (1H, dd, *J* = 13.2, 2.9, H-2), 6.10 (1H, s, H-6), 6.34 (1H, m, H-5'), 6.36 (1H, d, *J* =2.3 Hz, H-3'), 7.30 (1H, d, *J* = 8.2 Hz, H-6'); ^13^C NMR (125 MHz, acetone-*d_6_*) *δ* 18.2 (C-6''), 19.6 (C-9''), 26.3 (C-7''), 28.6 (C-1''), 32.8 (C-3''), 46.0 (C-3), 48.6 (C-2''), 56.2 (C-12''), 56.3 (C-11''), 75.9 (C-2), 93.7 (C-6), 103.8 (C-5'), 106.2 (C-4a), 108.1 (C-3'), 110.0 (C-8), 111.6 (C-10''), 118.9 (C-1'), 125.2 (C-4''), 128.9 (C-6'), 132.4 (C-5''), 150.2 (C-8''), 157.1 (C-2'), 159.9 (C-4'), 162.3 (C-5), 165.1 (C-8a), 165.3 (C-7), 194.3 (C-4).

Kushenol E (**3**): m.p. 160-162 °C; [α]_D_^25^ -15° (c 0.5, EtOH); HRESIMS *m/z* 423.1806 [M‒H]^‒^ (calcd for C_25_H_27_O_6_, 423.1808); ^1^H NMR (300 MHz, DMSO-*d_6_*) *δ* 1.54, 1.65 (6H, s, H-5'',5'''), 1.59, 1.76 (6H, s, H-4'',4'''), 2.66 (1H, dd, *J* = 4.0, 16.0 Hz, H-3'''), 3.18 (1H, dd, J = 12.0, 16.0 Hz, H-3''), 3.15 (2H, brs, H-1''), 3.34 (2H, br s, H-1'''), 5.05 (2H, br t, J = 7.0 Hz, H-2'',2'''), 5.52 (2H, dd, J = 4.0, 12.0 Hz, H-2), 6.26 (1H, dd, J = 8.0 Hz, H-5'), 6.34 (1H, d, J = 2.0 Hz, H-3'), 7.18 (1H, d, J = 8.0 Hz, H-6'); ^13^C NMR (75 MHz, DMSO-*d_6_*) *δ* 17.5 (C-5'''), 17.7 (C-5''), 20.8 (C-1''), 21.5 (C-1'''), 25.4 (C-4'''), 25.5 (C-4''), 39.7 (C-3), 73.7 (C-2), 101.8 (C-10), 102.3 (C-3'), 106.2 (C-5'), 107.2 (C-8), 107.7 (C-6), 115.8 (C-1'), 122.7 (C-2'',2'''), 127.7 (C-6'), 130.1 (C-3''), 130.3 (C-3'''), 155.6 (C-2'), 158.1 (C-4'), 158.3 (C-9), 158.4 (C-7), 161.4 (C-5), 197.5 (C-4).

Kushenol F (**4**): m.p. 131-132 °C; [α]_D_^25^ +6.5° (c 0.5, EtOH); HRESIMS *m/z* 423.1799 [M‒H]^‒^ (calcd for C_25_H_27_O_6_, 423.1808); ^1^H NMR (300 MHz, DMSO-*d_6_*) *δ* 1.58 (3H, s, H-7''), 1.62 (3H, s, H-10''), 1.70 (3H, s, H-6''), 2.00 (2H, m, H-3''), 2.47 (1H, m, H-2''), 2.59 (2H, m, H-1''), 2.74 (1H, dd, J = 4.0, 16.0 Hz, H-3b), 2.98 (1H, dd, J = 16.0, 12.0 Hz, H-3a), 4.58 (2H, br s, H-9''), 5.04 (1H, br t, J = 7.0 Hz, H-4''), 5.56 (1H, dd, J = 4.0, 12.0 Hz, H-2), 5.91 (1H, s, H-8), 6.34 (1H, d, J = 2.0 Hz, H-3'), 6.37 (1H, dd, J = 8.0 Hz, 2, H-5'), 7.31 (1H, d, J = 8.0 Hz, H-6'), 12.50 (s, OH-5).

(-)-Maackiain (**5**): Near yellow needles; m.p. 177-178 °C; [α]_D_^25^ -154.1° (c 0.5, acetone); HRESIMS *m/z* 285.0754 [M+H]^+^ (calcd for C_16_H_13_O_5_, 285.0763); ^1^H NMR (300 MHz, DMSO-*d_6_*) *δ* 3.64 (1H, t(dd), *J* = 10.6 Hz, H-6α), 3.51 (1H, m, H-6a), 4.24 (1H, dd, *J* = 10.9, 4.8 Hz, H-6β), 5.49 (1H, d, *J* = 7.0 Hz, H-11a), 5.93 (2H, dd, *J* = 13.0, 1.2 Hz, OCH_2_O), 6.26 (1H, d, *J* = 2.4 Hz, H-4), 6.51 (1H, s, H-10), 6.48 (1H, dd, *J* = 8.4, 2.4 Hz, H-2), 6.96 (1H, s, H-7), 7.25 (1H, d, *J* = 8.4 Hz, H-1); ^13^C NMR (75 MHz, DMSO-*d_6_*) *δ* 40.1 (C-6a), 65.7 (C-6α), 77.9 (C-11a), 93.2 (C-10), 100.9 (OCH_2_O), 102.8 (C-4), 105.3 (C-7), 109.6 (C-2), 111.2 (C-11b), 118.4 (C-6b), 131.9 (C-1), 141.0 (C-8), 147.4 (C-9), 153.7 (C-10a), 156.3 (C-4a), 158.7 (C-3).

Noranhydroicaritin (**6**): yellow powder; m.p. 112-115 °C; HRESIMS *m/z* 353.1030 [M‒H]^‒^ (calcd for C_20_H_18_O_6_, 353.1025); ^1^H NMR (500 MHz, acetone-*d_6_*) *δ* 1.68 (3H, s, H-5''), 3.42 (2H, m, H-1''), 4.53 (3H, s, H-4''), 5.15 (1H, m, H-2''), 6.22 (1H, s, H-6), 6.90 (4H, d, *J* = 2.85 Hz, H-3', 5'), 8.05 (4H, d, *J* = 2.85 Hz, H-2', 6'); ^13^C NMR (125 MHz, acetone -*d_6_*) *δ* 18.5 (C-4''), 22.6 (C-1''), 26.2 (C-5''), 99.2 (C-6), 104.6 (C-4a), 107.6 (C-8), 116.8 (C-3', 5'), 124.0 (C-2), 124.1 (C-2''), 130.8 (C-2', 6'), 132.5 (C-1'), 136.9 (C-3''), 147.4 (C-3), 155.5 (C-4'), 160.3 (C-8a), 160.5 (C-5), 162.5 (C-7), 177.2 (C-4).

(-)-4-Hydroxy-3-methoxy-8,9-methylenedioxypterocarpan (**7**): m.p. 172-173 °C; [α]_D_^25^ -154.1° (c 0.5, acetone); HRESIMS *m/z* 315.0841 [M+H]^+^ (calcd for C_17_H_15_O_6_, 315.0869); ^1^H NMR (300 MHz, DMSO-*d_6_*) *δ* 3.58 (1H, ddd, *J* = 5.0, 7.0, 11.0 Hz, H-6a), 3.62 (1H, t, *J* = 4.5, 11.0 Hz, H-6β), 3.76 (s, 3-OCH_3_), 4.27 (1H, dd, *J* = 4.5, 11.0 Hz, H-6α), 5.58 (1H, d, *J* = 7.0 Hz, H-11a), 5.92 (OCH_2_O), 6.51 (1H, s, H-10), 6.68 (1H, d, *J* = 2.5, 8.5 Hz, H-2), 6.87 (1H, s, *J* = 8.5 Hz, H-1), 6.98 (1H, s, H-7); ^13^C NMR (75 MHz, DMSO-*d_6_*) *δ* 39.8 (C-6a), 56.0 (3-OCH_3_), 66.1 (C-6α), 78.1 (C-11a), 93.1 (C-10), 100.9 (OCH_2_O), 105.3 (C-7), 106.0 (C-2), 114.3 (C-11b), 118.3 (C-6b), 120.1 (C-1), 134.6 (C-4), 144.4 (C-3), 141.0 (C-8), 147.4 (C-4a), 148.2 (C-9).

Sophoraflavanone B (**8**): m.p. 202-203 °C; [α]_D_^25^ -25° (c 0.1, EtOH); HRESIMS *m/z* 339.1228 [M‒H]^‒^ (calcd for C_20_H_19_O_5_, 339.1232); ^1^H NMR (300 MHz, DMSO-*d_6_*) d 1.54 (3H, s, H-4''), 1.58 (3H, s, H-5''), 2.71 (m, H-3), 3.08 (br d, *J* = 7.9 Hz, H-1''), 5.08 (br t, *J* = 7.9 Hz, H-2''), 5.42 (q, *J* = 3.5, 12.1 Hz, H-2), 5.97 (s, H-6), 6.78 (d, *J* = 8.8 Hz, H-3',5'), 7.30 (d, *J* = 8.8 Hz, H-2',6'), 9.53 (4'-OH), 10.71 (7-OH), 12.10 (5-OH); ^13^C NMR (75 MHz, DMSO-*d_6_*) *δ* 17.5 (C-4''), 21.2 (C-1''), 25.5 (C-5''), 41.8 (C-3), 78.3 (C-2), 95.3 (C-8), 101.8 (C-10), 106.9 (C-6), 115.1 (C-3',5'), 122.7 (C-2''), 128.0 (C-2',6'),, 129.2 (C-1'), 130.1 (C-3''), 157.5 (C-4'), 159.7 (C-9), 161.1 (C-5), 164.3 (C-7), 196.6 (C-4).


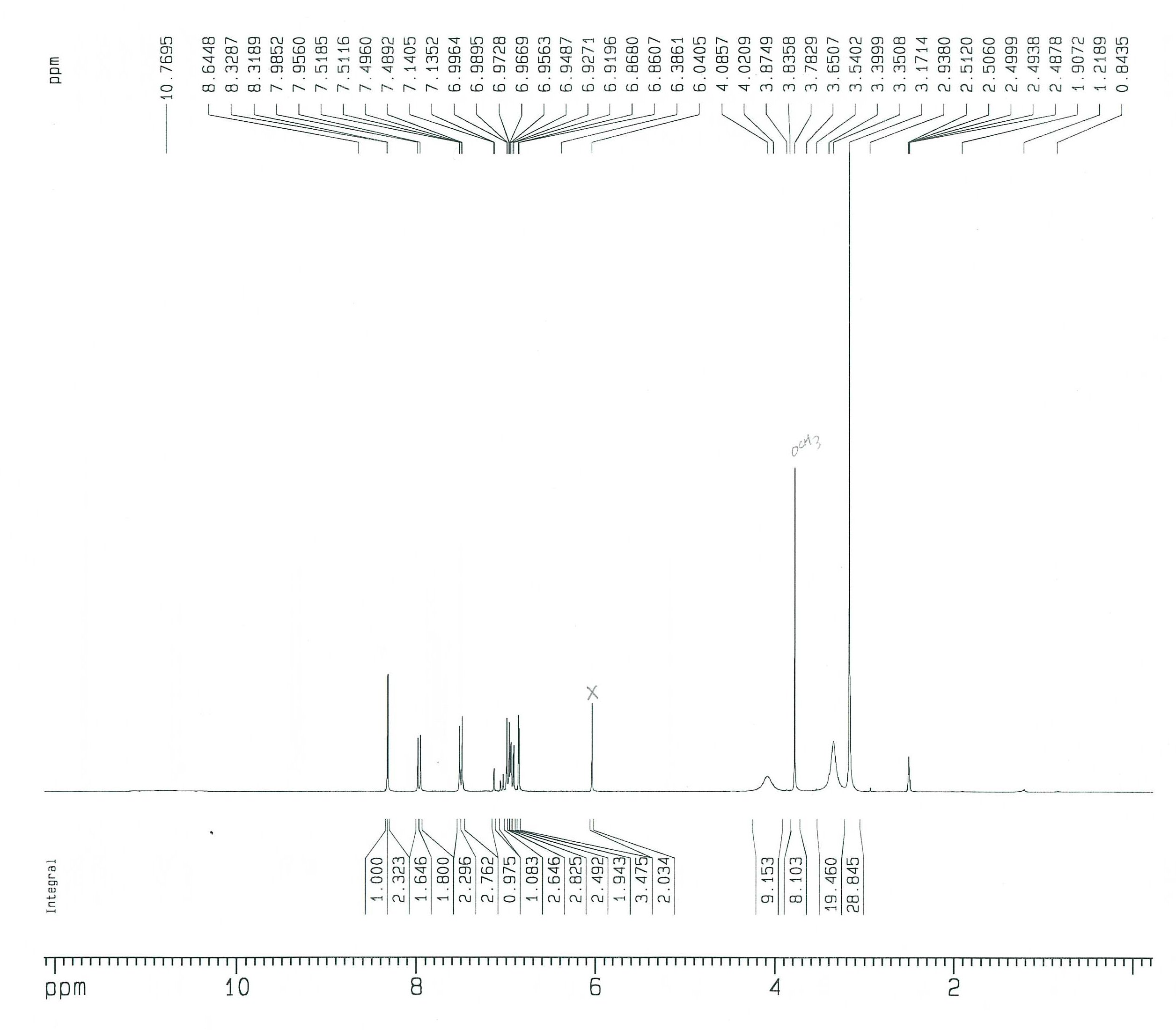


**Figure S3-1.** ^1^H-NMR spectrum of formononetin **1** (400 MHz, DMSO-*d_6_*).


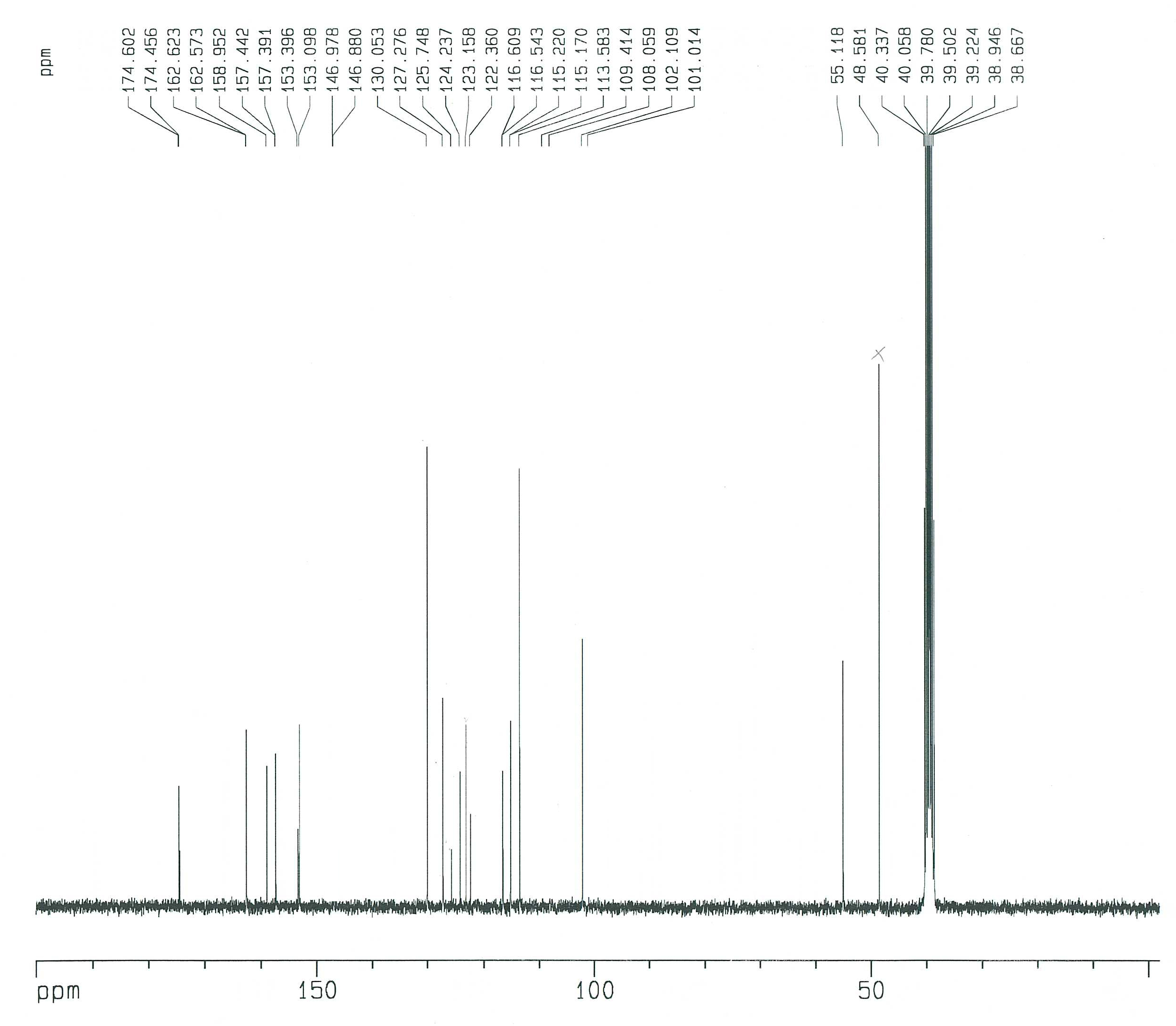


**Figure S3-2.** ^13^C-NMR spectrum of formononetin **1** (100 MHz, DMSO-*d_6_*).


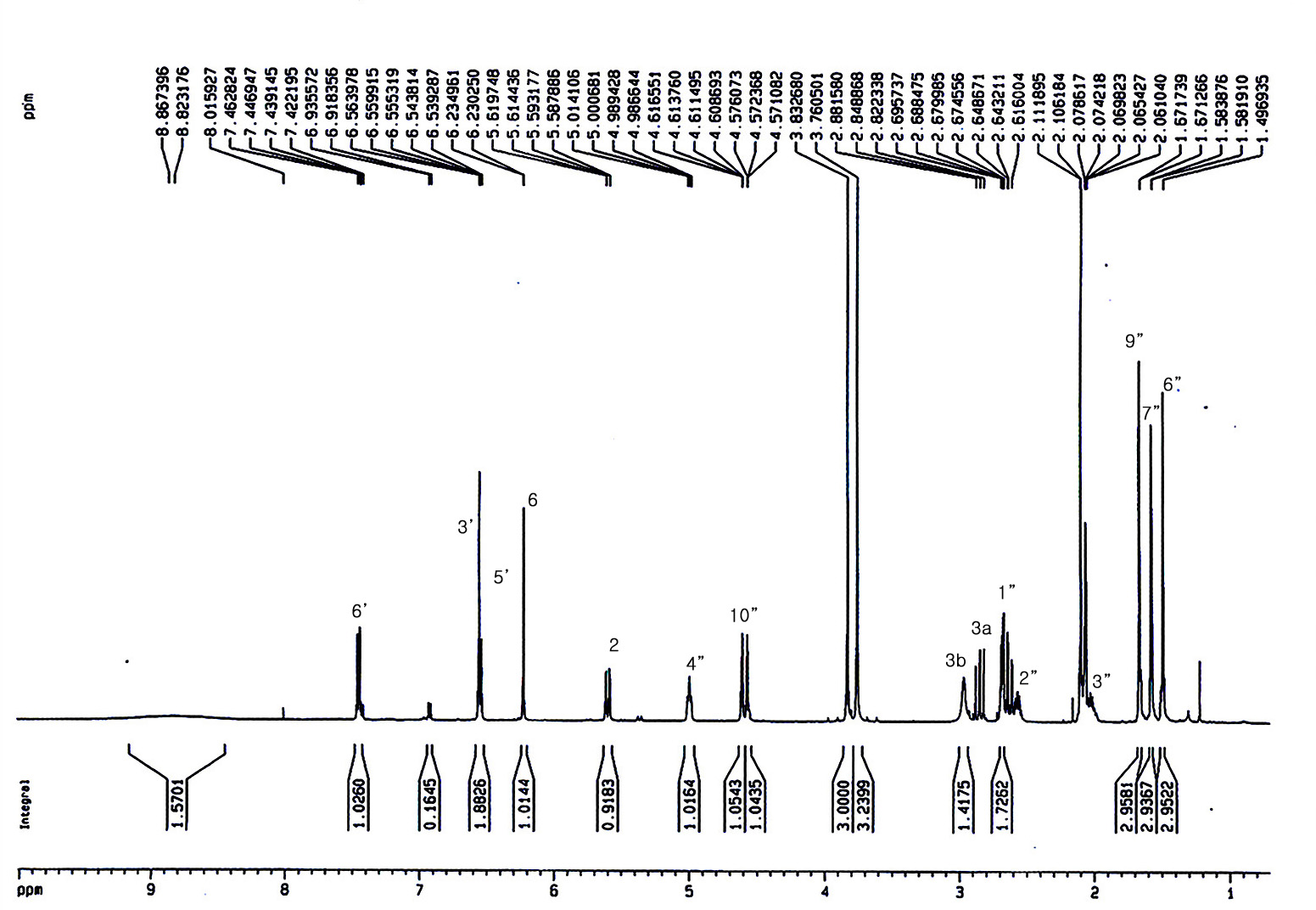


**Figure S-3-3.** ^1^H-NMR spectrum of (2S)-2'-methoxy kurarinone **2** (400 MHz, DMSO-*d_6_*).


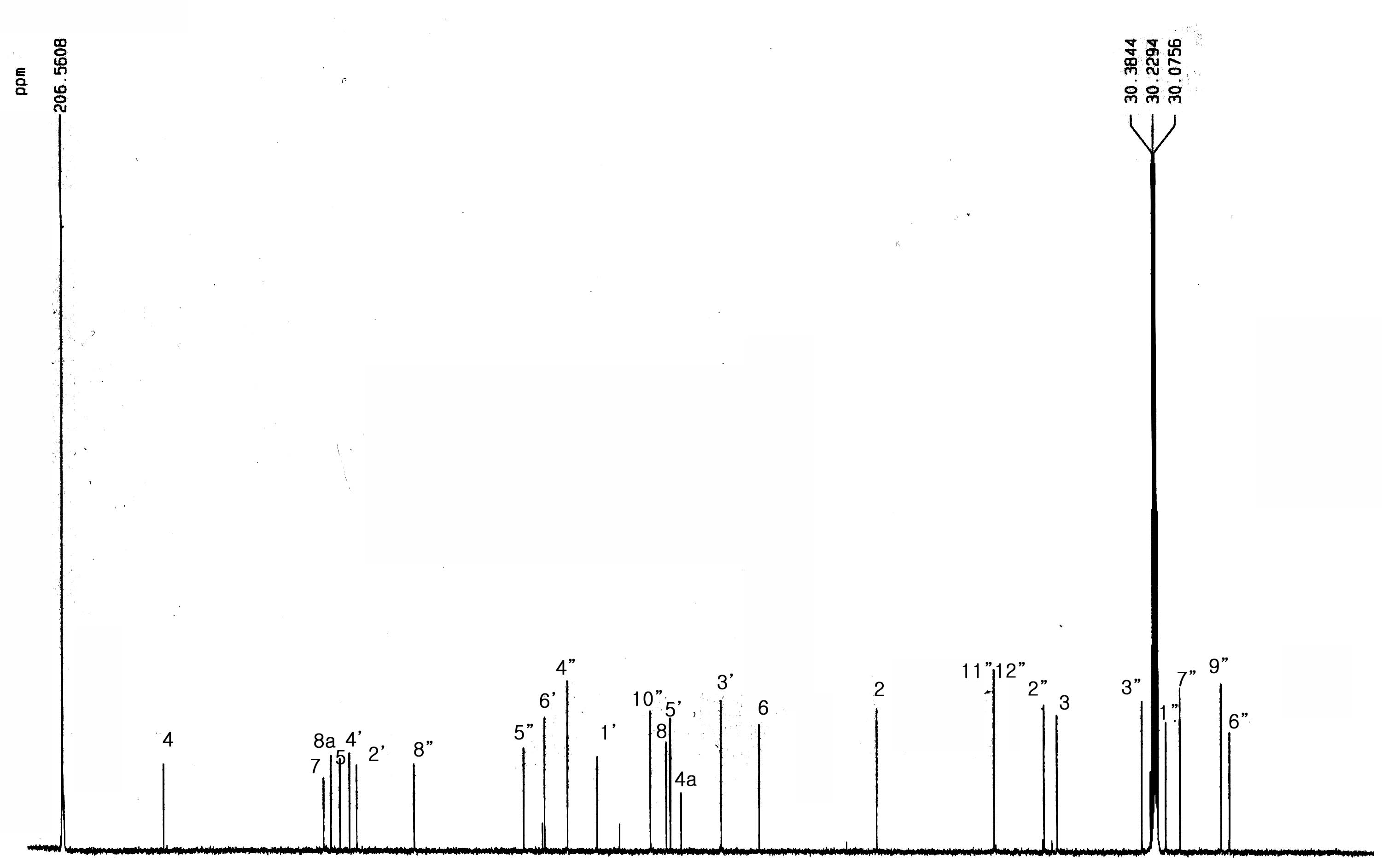


**Figure S3-4.** ^13^C-NMR spectrum of (2S)-2'-methoxy kurarinone **2** (100 MHz, DMSO-*d_6_*).


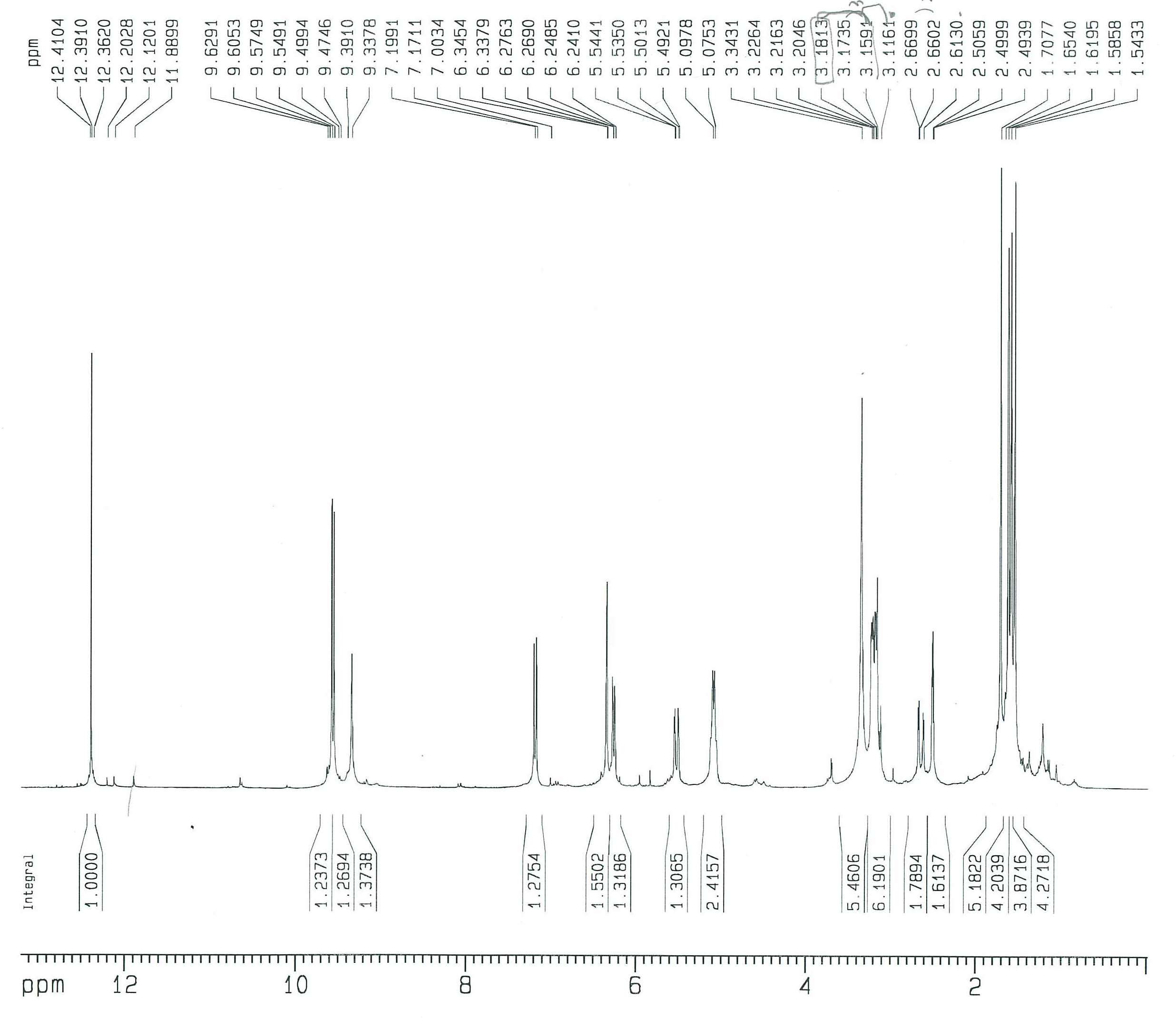


**Figure S3-5.** ^1^H-NMR spectrum of kushenol E **3** (400 MHz, DMSO-*d_6_*).


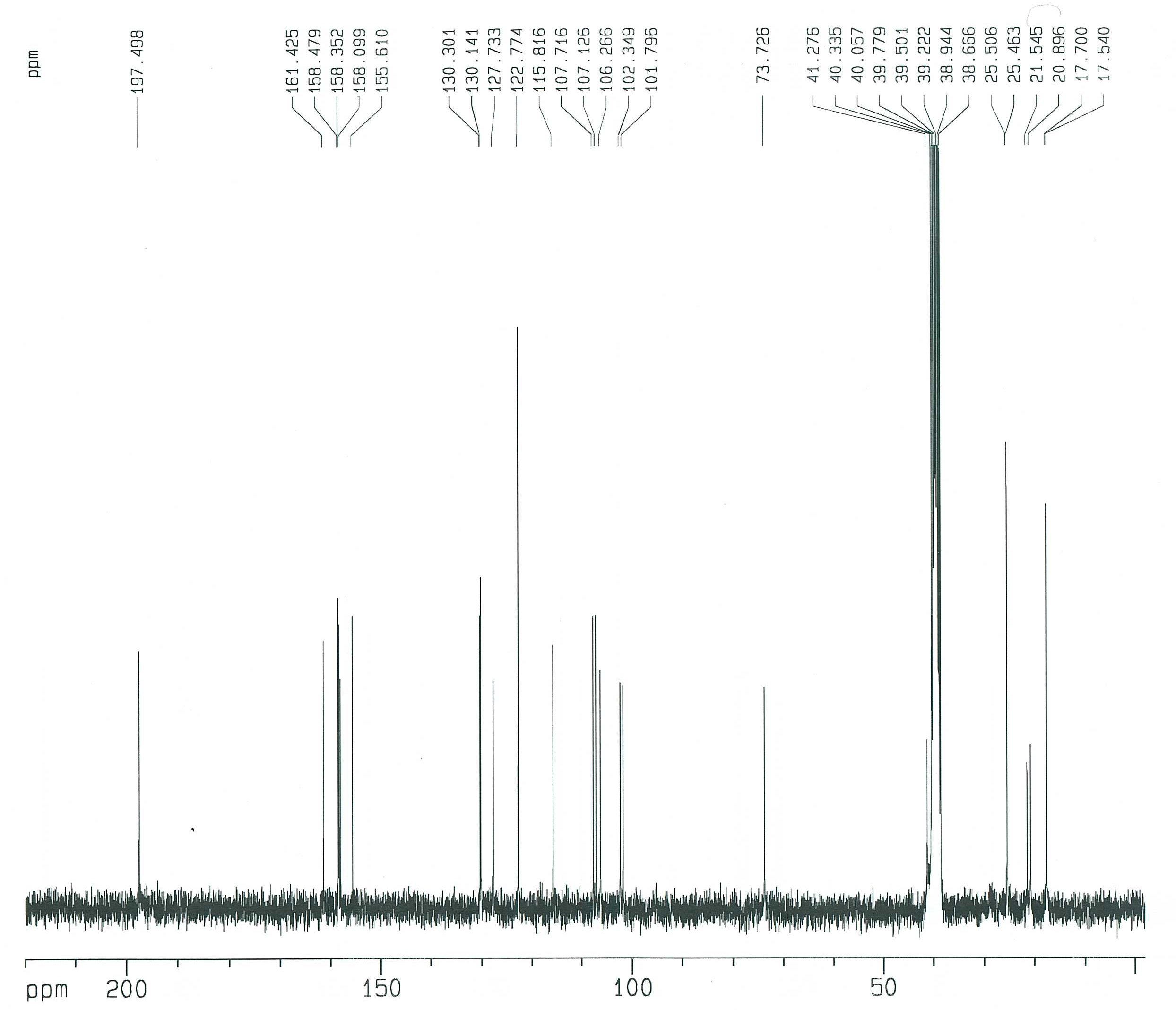


**Figure S3-6.** ^13^C-NMR spectrum of kushenol E **3** (100 MHz, DMSO-*d_6_*).


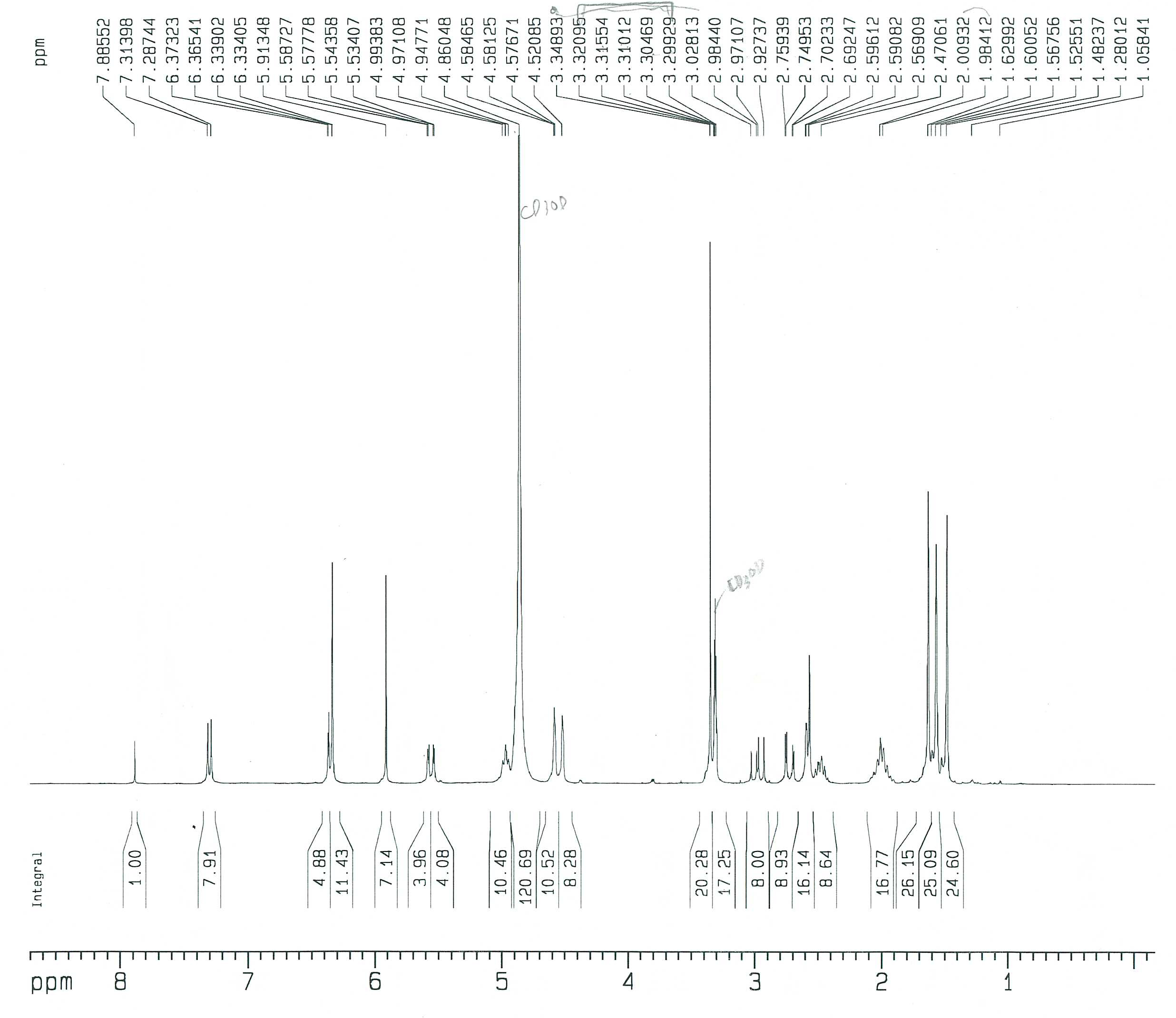


**Figure S3-7.** ^1^H-NMR spectrum of kushenol F **4** (400 MHz, DMSO-*d_6_*).


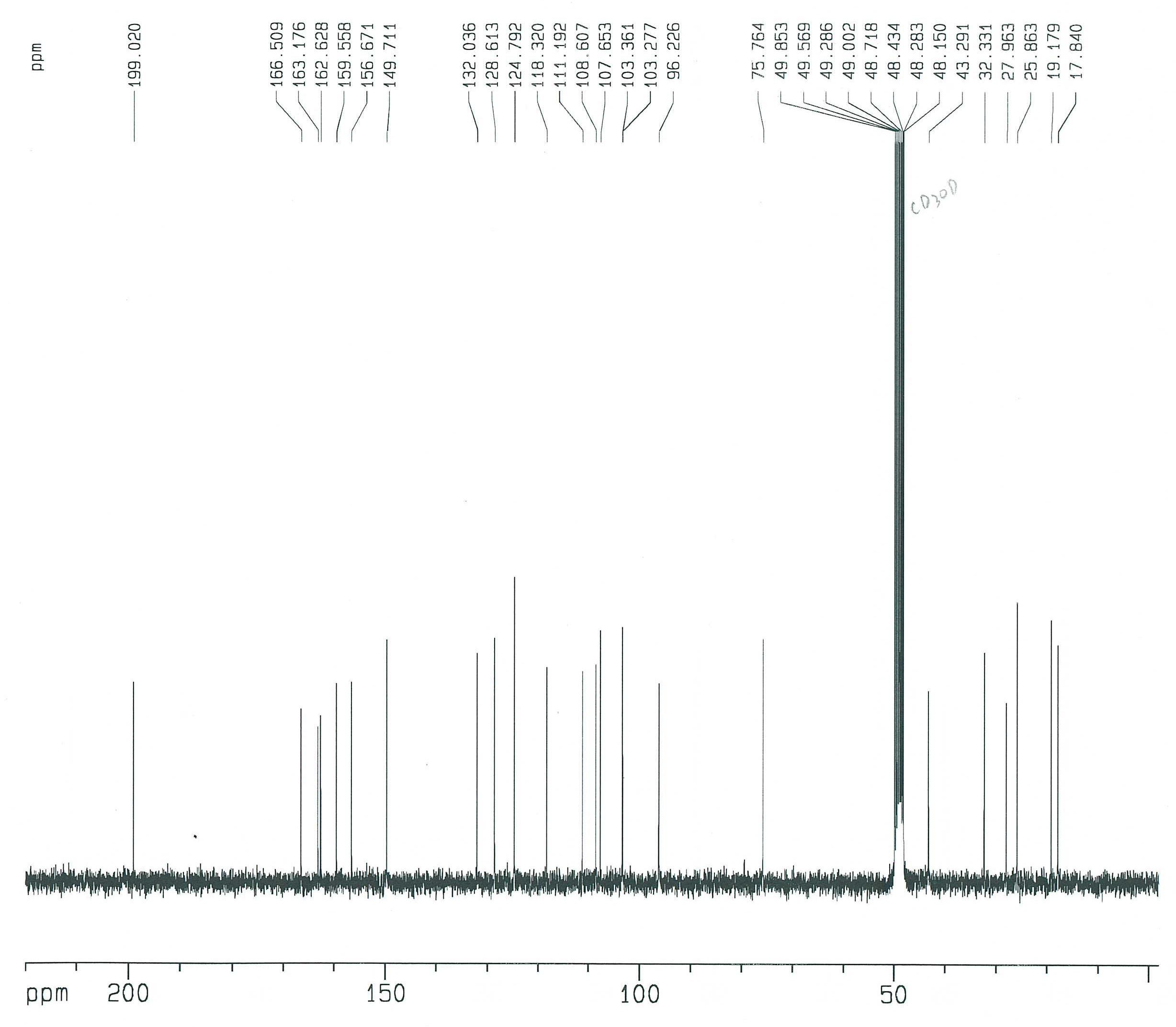


**Figure S3-8.** ^13^C-NMR spectrum of kushenol F **4** (100 MHz, DMSO-*d_6_*).


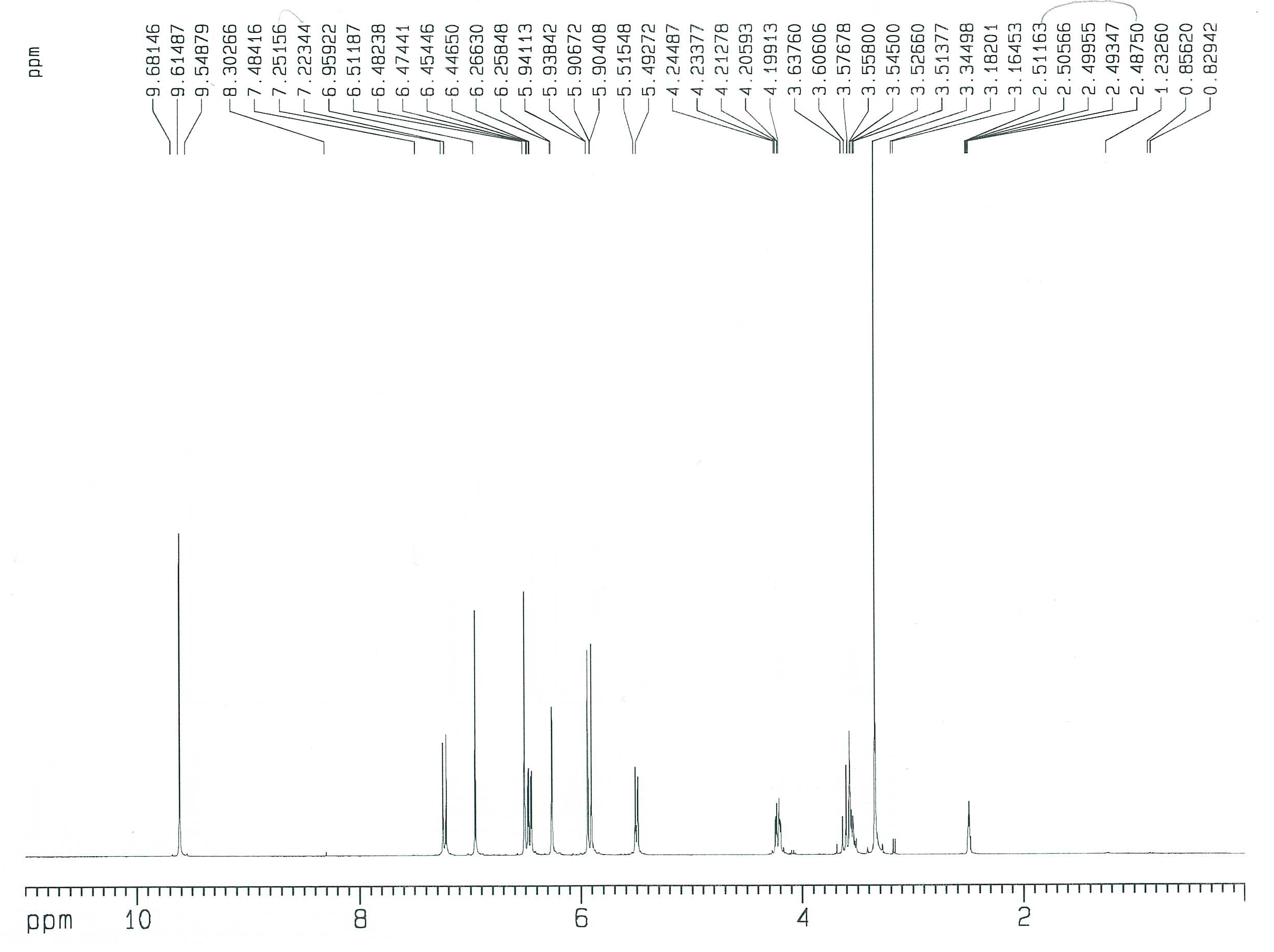


**Figure S3-9.** ^1^H-NMR spectrum of (-)-maackiain **5** (400 MHz, DMSO-*d_6_*).


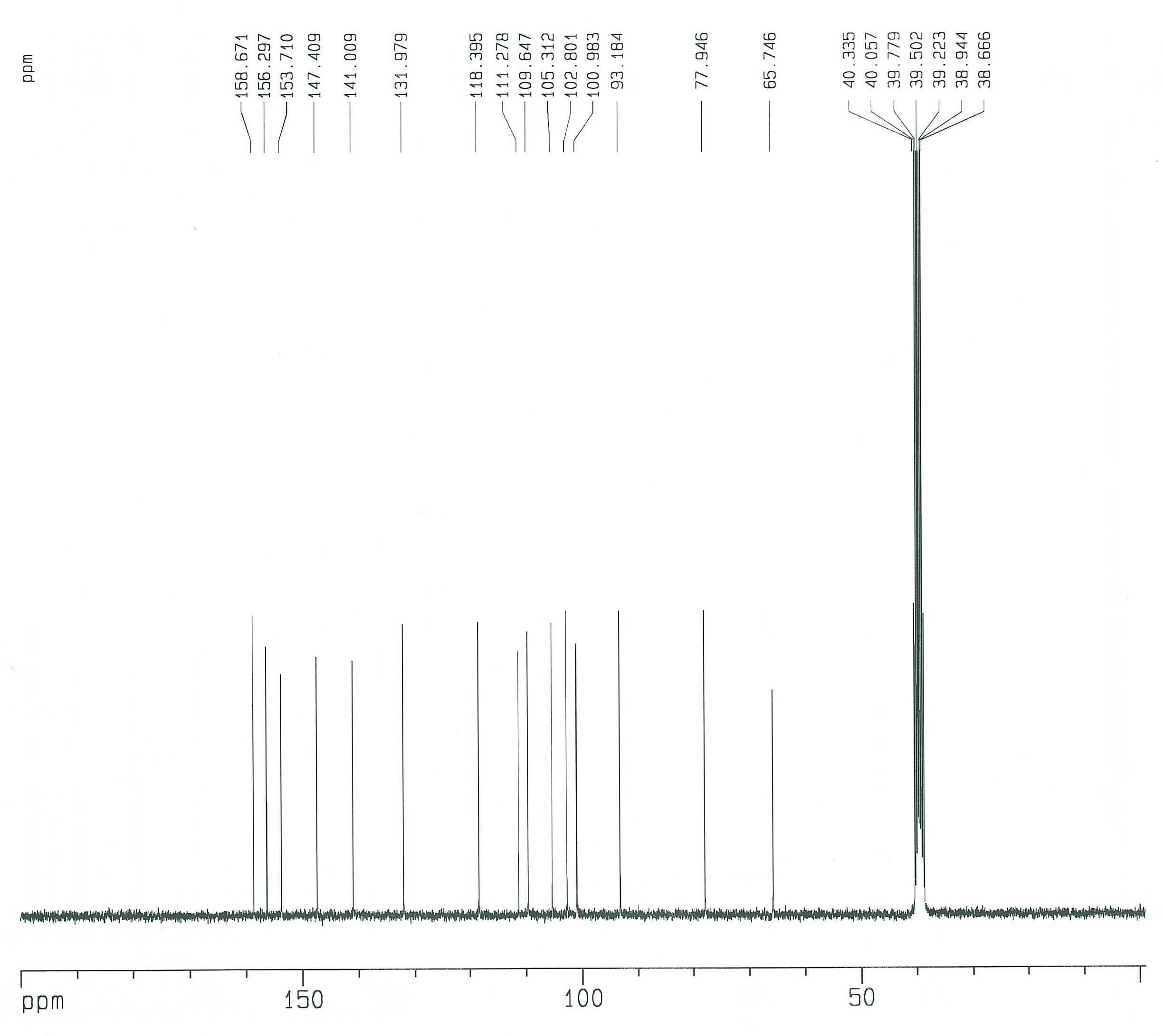


**Figure S3-10.** ^13^C-NMR spectrum of (-)-maackiain **5** (100 MHz, DMSO-*d_6_*).


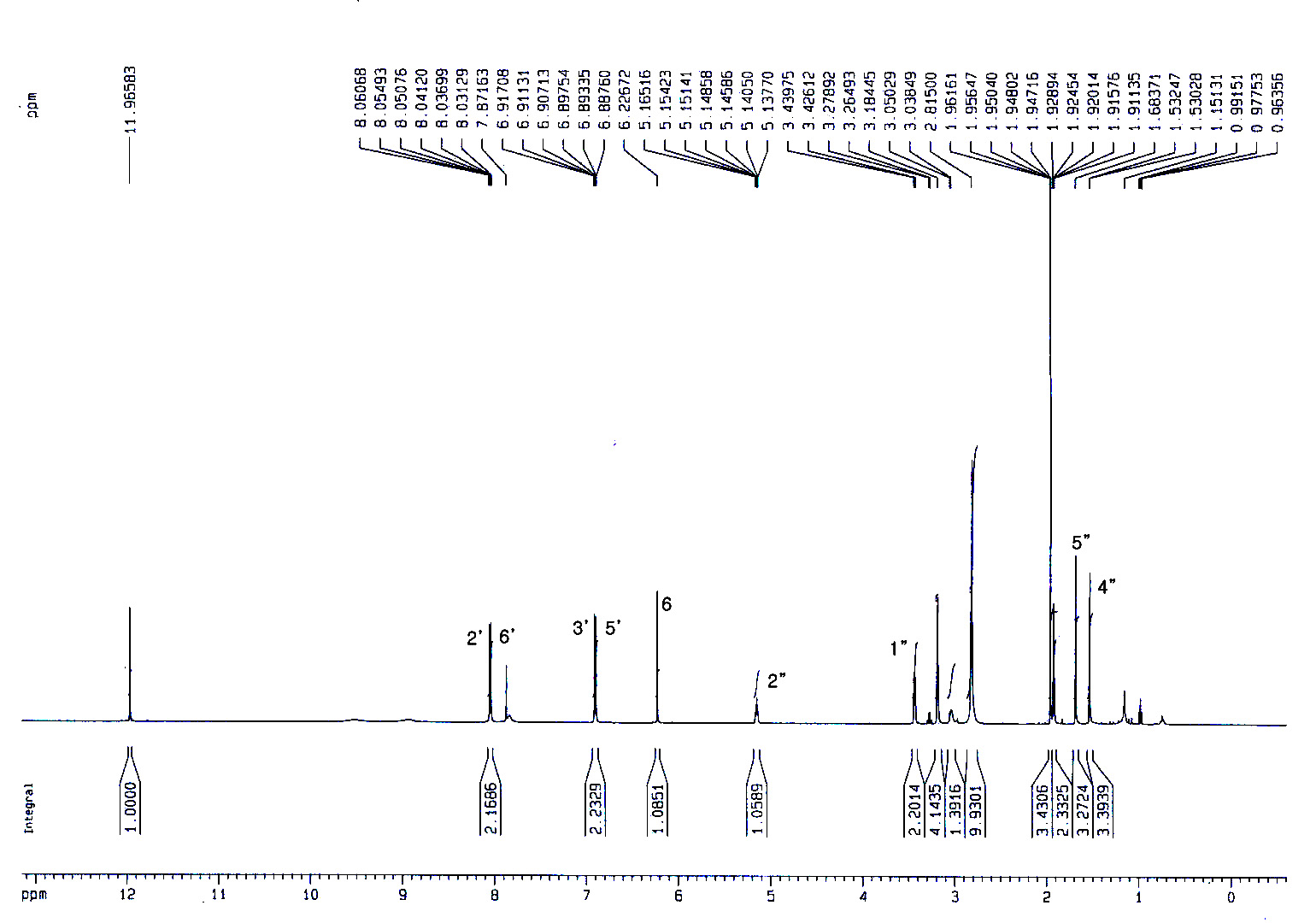


**Figure S3-11.** ^1^H-NMR spectrum of noranhydroicaritin **6** (400 MHz, DMSO-*d_6_*).


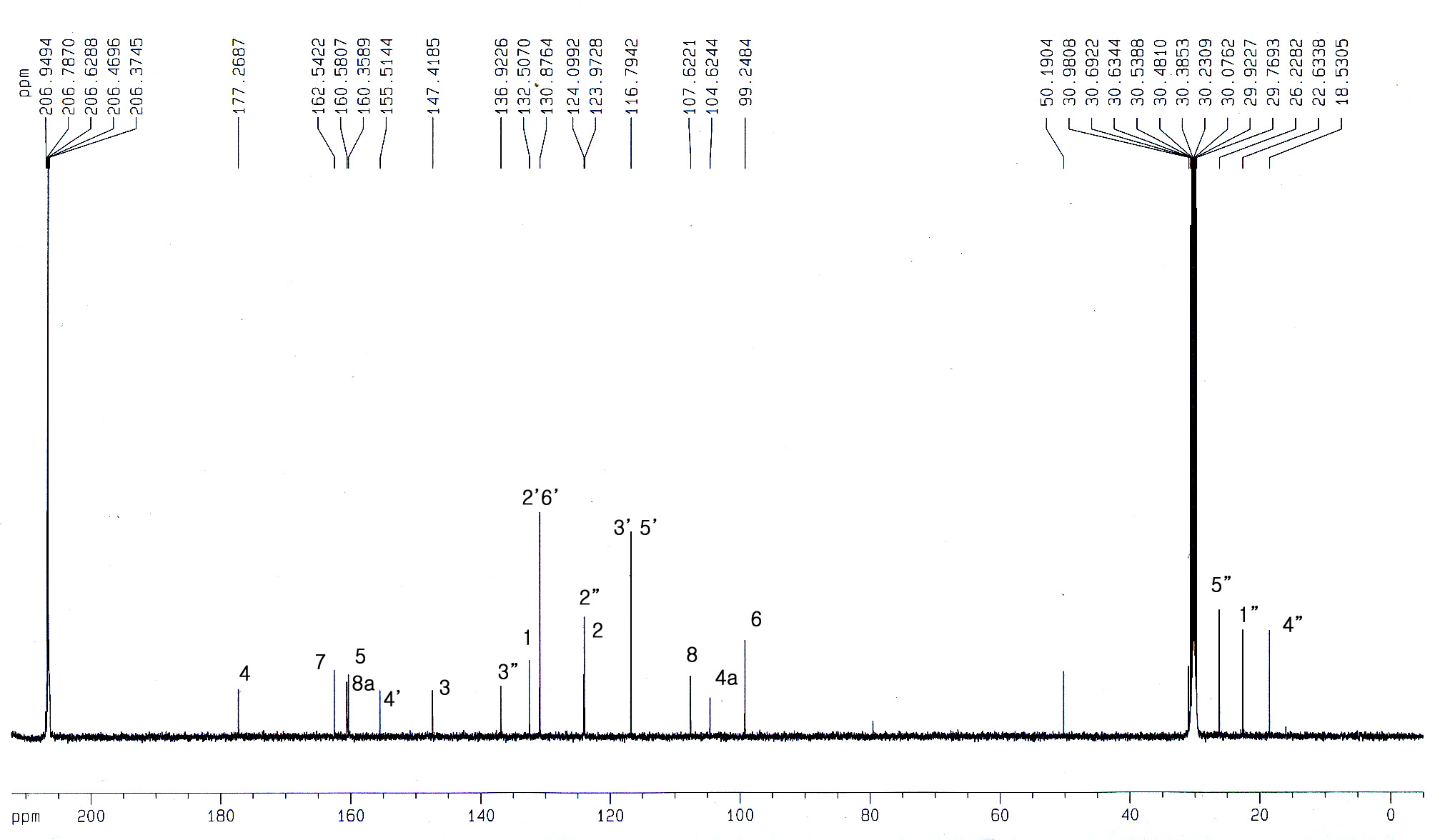


**Figure S3-12.** ^13^C-NMR spectrum of noranhydroicaritin **6** (100 MHz, DMSO-*d_6_*).


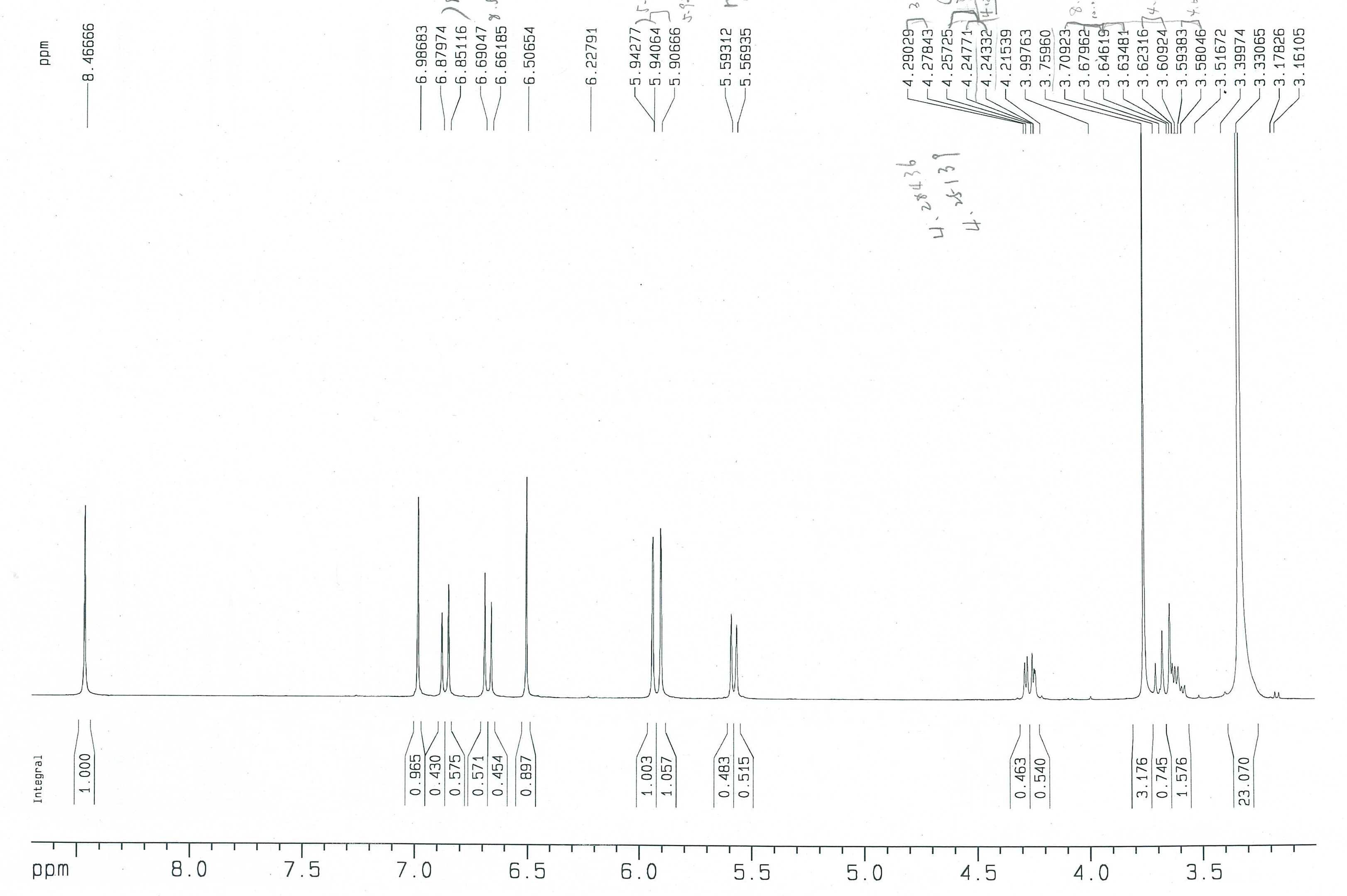


**Figure S3-13.** ^1^H-NMR spectrum of (-)-4-hydroxy-3-methoxy-8,9-methylenedioxypterocarpan **7** (400 MHz, DMSO-*d_6_*).

**
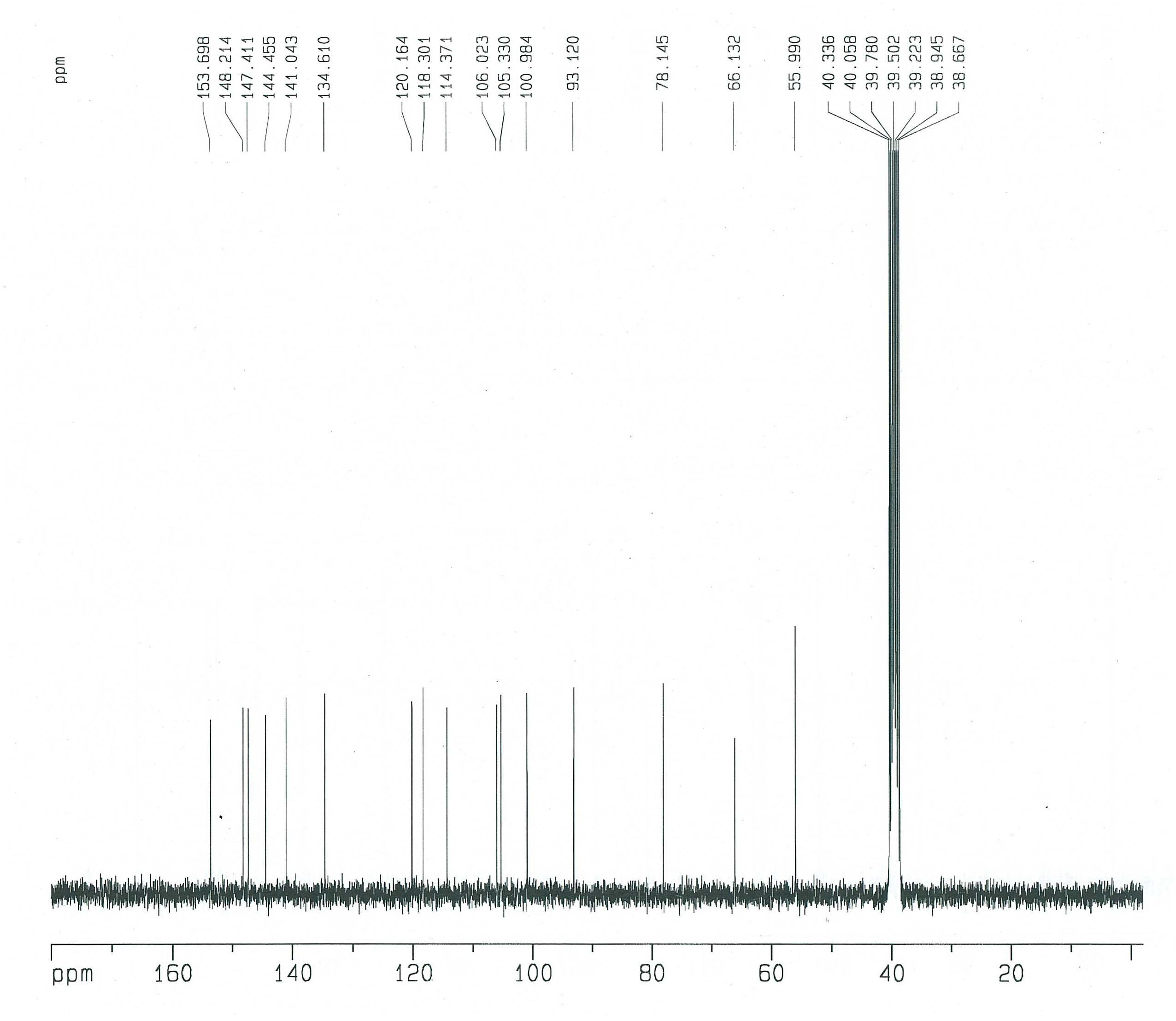
**

**Figure S3-14.** ^13^C-NMR spectrum of (-)-4-hydroxy-3-methoxy-8,9-methylenedioxypterocarpan **7** (100 MHz, DMSO-*d_6_*).


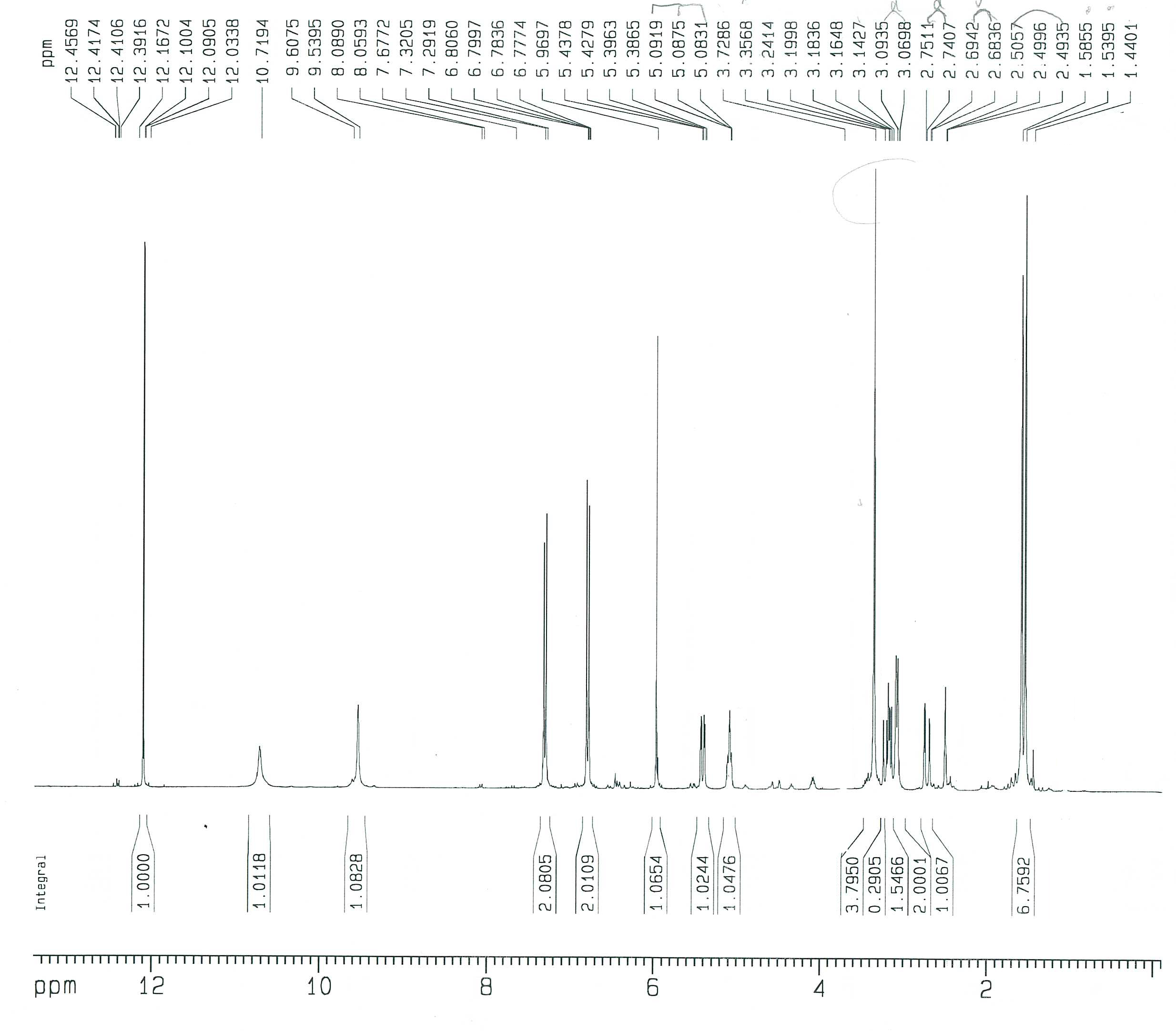


**Figure S3-15.** ^1^H-NMR spectrum of sophoraflavanone B **8** (400 MHz, DMSO-*d_6_*).


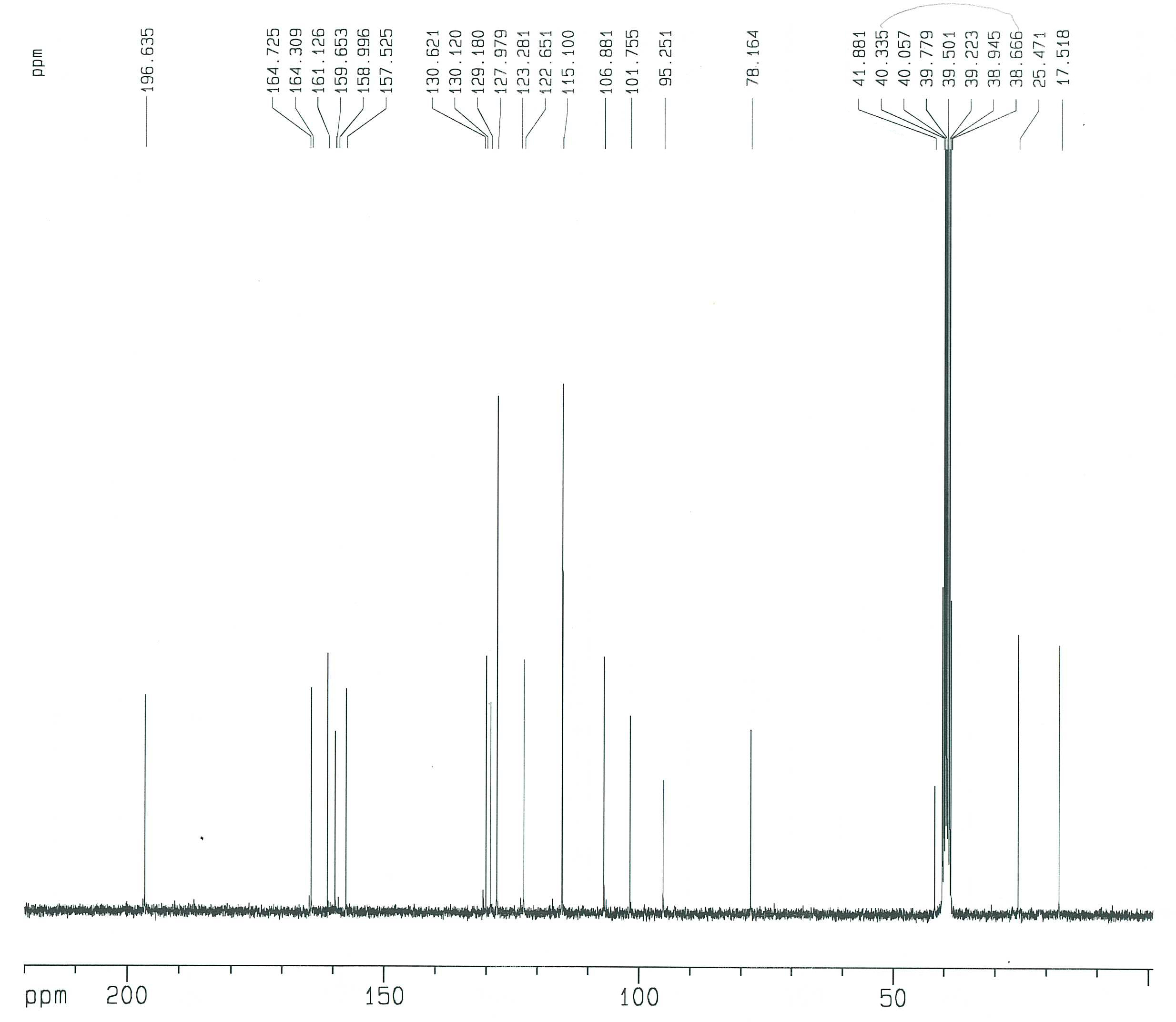


**Figure S3-16.** ^13^C-NMR spectrum of sophoraflavanone B **8** (100 MHz, DMSO-*d_6_*).
